# Comprehensive analysis of partial epithelial mesenchymal transition‐related genes in hepatocellular carcinoma

**DOI:** 10.1111/jcmm.16099

**Published:** 2020-11-20

**Authors:** Yu Lei, Wei Yan, Zhuoying Lin, Jingmei Liu, Dean Tian, Ping Han

**Affiliations:** ^1^ Department of Gastroenterology Tongji Hospital of Tongji Medical College Huazhong University of Science and Technology Wuhan China

**Keywords:** competing endogenous RNA, hepatocellular carcinoma, partial epithelial mesenchymal transition, prognosis

## Abstract

Increasing evidence has revealed that cancer cells undergoing an intermediate state, partial epithelial mesenchymal transition (p‐EMT), tend to metastasize rather than complete EMT. We performed a comprehensive analysis of E‐cadherin and 25 p‐EMT‐related genes in HCC to explore the roles and regulatory mechanisms of them in HCC. We analysed E‐cadherin and 25 p‐EMT‐related genes in HCC and constructed an mRNA‐miRNA‐lncRNA ceRNA subnetwork containing p‐EMT‐related genes by bioinformatic approaches. IHC was used to identify the protein expression of key p‐EMT‐related genes, P4HA2, ITGA5, MMP9, MT1X and SPP1. Complete EMT is not necessary for HCC progression. Overexpression of P4HA2, ITGA5, MMP9, SPP1 and down‐regulation of MT1X were found in HCC tissues, which were significantly associated with poor prognosis of HCC patients. By means of stepwise reverse prediction and validation from mRNA to lncRNA, an mRNA‐miRNA‐lncRNA ceRNA subnetwork correlated with HCC prognosis was identified by expression and survival analysis. This study implied that key p‐EMT‐related genes P4HA2, ITGA5, MMP9, MT1X, SPP1 could be prognostic biomarkers and potential targets of therapy for HCC patients. We constructed an mRNA‐miRNA‐lncRNA subnetwork containing p‐EMT‐related genes successfully, among which each component might be utilized as a prognostic biomarker of HCC.

## 1 INTRODUCTION

Hepatocellular carcinoma (HCC) is one of the most common cancers worldwide and listed as the third leading cause of cancer‐related mortality.^1^ The long‐term prognosis of patients with HCC after hepatectomy remains poor because of frequent metastasis.^2^ Though efforts have been made in investigating the mechanisms of the HCC initiation and progression, detailed mechanisms of HCC pathogenesis remain largely unclear. Promising biomarkers of HCC urgently need to be identified for HCC diagnosis and therapy improvement.

It is widely acknowledged that epithelial mesenchymal transition (EMT) plays a vital role in invasion and metastasis in diverse types of cancer, including HCC.^3^, ^4^ Cancer cells during EMT lose their epithelial features and gain fully mesenchymal phenotypes, known as complete EMT.^5^ Down‐regulation of epithelial markers such as E‐cadherin (also named CDH1), and up‐regulation of mesenchymal markers such as vimentin are considered as hallmarks of EMT and growing studies have shown identify the importance of EMT in HCC progression.^6^, ^7^ Recently, an intermediate state of EMT named partial‐EMT(p‐EMT), during which cancer cells exhibit both mesenchymal and epithelial features, has attracted more and more attention. Recent studies have found that metastatic cancer cells express a certain level of E‐cadherin, and cancer cells in p‐EMT state have an intense trend to adapt to the metastatic microenvironment a higher risk of metastasis.^8^ Our understanding of p‐EMT programme was enhanced using sophisticated techniques such as lineage tracing in patient‐derived xenografts, genetically engineered mouse models (GEMM), single‐cell RNA sequence, fluorescent‐activated cell sorting (FACS).^9^ A single‐cell transcriptomic analysis of head and neck squamous cell carcinoma (HNSCC) has identified several representative genes in p‐EMT programme in malignancy including 15 common p‐EMT‐related genes (SERPINE1, TGFBI, MMP10, LAMC2, P4HA2, PDPN, ITGA5, LAMA3, CDH13, TNC, MMP2, EMP3, INHBA, LAMB3 and VIM) and 10 variable p‐EMT‐related genes (THBS2, CXCL13, FN1, MMP3, MMP9, RAB25, MT1X, GPX3, SPP1 and MXD1).^10^ The 25 p‐EMT‐related genes are predominantly abundant in extracellular matrix and cell membrane. Previous studies have identified the presence of p‐EMT programme in HCC cells.^11^, ^12^ However, the nature of p‐EMT‐related genes in HCC remains unknown. Although several hallmarks, such as p53 and WNT signalling, were reported to be closely correlated with HCC, little evidence demonstrated their direct role as markers of EMT process. Due to the lack of using sophisticated techniques in p‐EMT programme in HCC, we performed analysis of 25 p‐EMT‐related genes identified in HNSCC previously, all of which were confirmed to be involved in EMT programme.

A competing endogenous RNA (ceRNA) hypothesized that non‐coding RNA (ncRNA), including long non‐coding RNA (lncRNA), can gain cross‐talk with mRNAs by competitively binding to shared miRNAs and then form a regulatory network was proposed by Salmena et al^13^ Accumulating evidence has indicated that the lncRNA‐miRNA‐mRNA ceRNA network may play an important role in cancer metastasis including HCC.^14^ Recently, a lncRNA‐miRNA‐mRNA ceRNA network associated with diagnosis and prognosis of HCC has been established.^15^ Nevertheless, understanding of mRNA‐miRNA‐lncRNA ceRNA networks containing p‐EMT‐related genes correlated significantly with prognosis of HCC remain extremely limited and need to be explored.

In our study, we performed a comprehensive analysis of E‐cadherin in HCC. Then, we analysed the expression patterns, prognostic values, gene mutations, mutual interactions, correlations with each other of 25 p‐EMT‐related genes and identified key genes after comprehensive consideration of expression and prognostic roles of p‐EMT‐related genes using online databases. We selected key upstream miRNAs and lncRNAs regulating the key genes to construct an mRNA‐miRNA‐lncRNA ceRNA subnetwork associated with prognosis of HCC. Furthermore, we identified protein expression levels of the selected key p‐EMT‐related genes in HCC tissues and adjacent nontumour liver tissues by immunohistochemistry (IHC) staining. They may also serve as potential biomarkers for diagnostic and therapeutic improvement of HCC in the future.

## 2 MATERIAL AND METHODS

### 2.1 HCCDB database analysis

HCCDB (http://lifeome.net/database/hccdb/home.html)is an integrative molecular database of HCC containing 15 public HCC datasets, such as Gene Expression Omnibus (GEO), Liver Hepatocellular Carcinoma Project of The Cancer Genome Atlas (TCGA‐LIHC) and Liver Cancer‐RIKEN, JP Project from International Cancer Genome Consortium (ICGC‐LIRI‐JP).^16^ HCCDB database was utilized to analyse the expression patterns of E‐cadherin and 25 p‐EMT‐related genes in HCC (Figure 1 & Figures S9‐S33), as well as other types of cancers in TCGA. Prognostic value of E‐cadherin was also explored by HCCDB.

**FIGURE 1:**
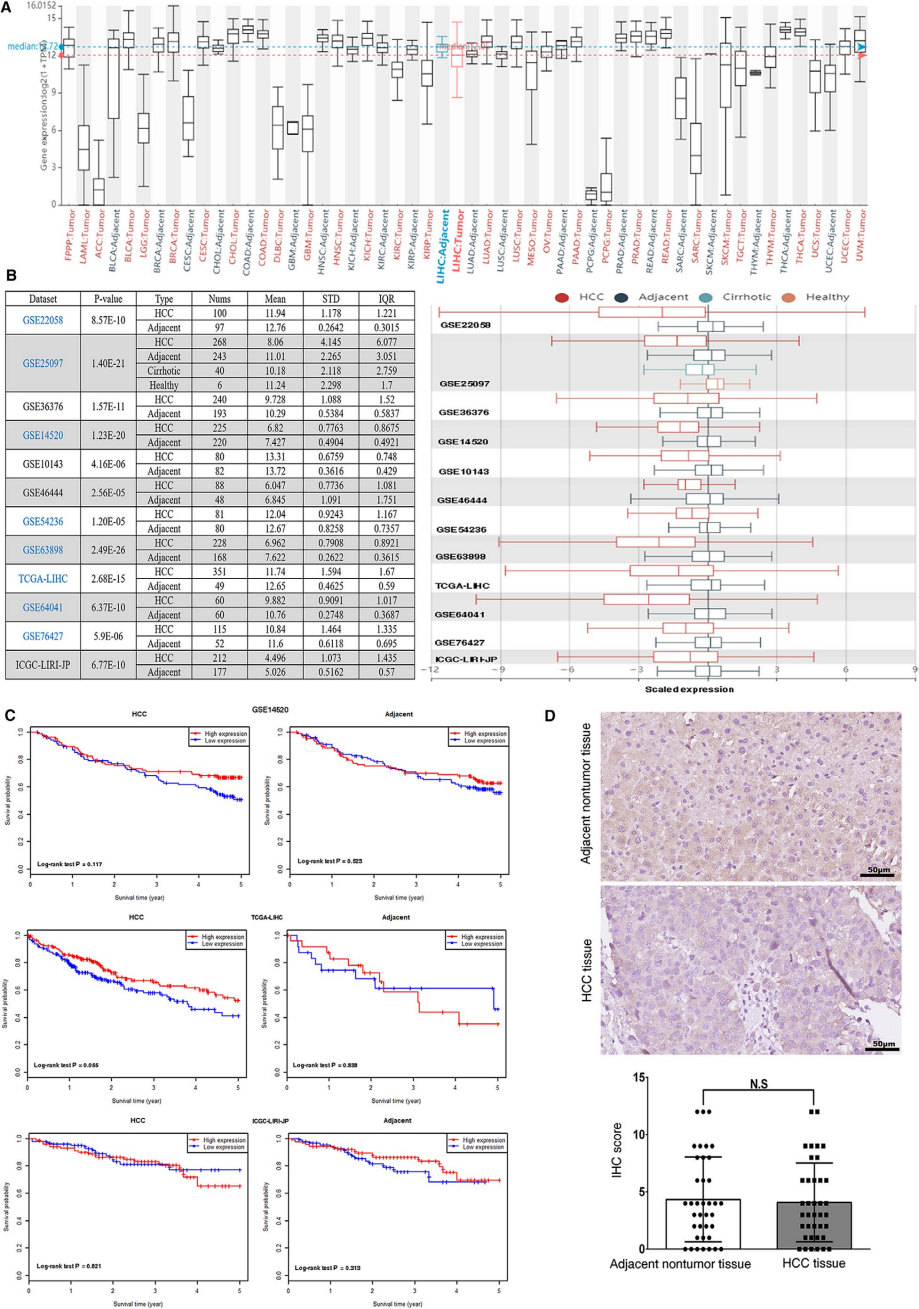
Comprehensive analysis of E‐cadherin in HCC. A, The mRNA expression levels of E‐cadherin across multiple cancer types in TCGA. B, Chart and plot showing the expression of E‐cadherin in HCC tissues and the adjacent normal tissues in HCCDB. C, The correlation between mRNA expression levels of E‐cadherin and survival of HCC patients in three datasets. D, Representative IHC images and IHC score of E‐cadherin in HCC tissues and adjacent nontumour tissues

### 2.2 Patients and IHC assay

HCC samples and the corresponding adjacent nontumour liver tissues were collected from adult patients with HCC who underwent curative resection at the Tongji Hospital of Tongji Medical College (Wuhan, China). A preoperative clinical diagnosis of HCC was based on the diagnostic criteria of the American Association for the Study of Liver Diseases and haematoxylin & eosin staining of the samples was also performed. All of the samples were selected with distinctive pathologic diagnosis and none of the patients received any preoperative chemotherapy or radiotherapy. These tissues were stained for E‐cadherin (Cell Signaling Technology, 3195), MMP10 (Abclonal, A3033), P4HA2 (Abcam, ab233197), ITGA5 (Abcam, ab150361), MMP9 (Cell Signaling Technology, 13667), MT1X (Proteintech, 17172‐1‐AP) and SPP1 (Abclonal, A1499) expression. Furthermore, negative control for IHC staining was shown in Figure S8. IHC staining was performed using the Dako Envision Plus System (Dako) according to the manufacturer's instructions. The IHC staining intensity was scored as 0 (negative); 1 (weak); 2 (medium); 3 (strong). The percentage of positive cells was scored from 0 to 4 (0%, 1%‐25%, 26%‐50%, 51%‐75%, 76%‐100%). Overall score ranging from 0 to 12 was calculated by multiplying the above two scores, resulting in a negative (0‐3) staining or a positive (4‐12) staining for each example.

### 2.3 UALCAN database analysis

UALCAN (http://ualcan.path.uab.edu) is a newly developed interactive web resource for 31 cancer types from TCGA database.^17^ In this study, UALCAN database, containing 371 primary HCC samples and 50 normal samples, was used to analyse mRNA expression levels of p‐EMT‐related genes in HCC. In addition, correlations between genes and clinical characteristics were investigated in UALCAN database. Transcript per million <1 was excluded due to the extremely low value. *P* value <.05 was considered as statistically significant.

### 2.4 Oncomine database analysis

Oncomine (https://www.oncomine.org/) is an integrated online cancer microarray database.^18^ In this study, Oncomine was used to analyse mRNA expression levels of the 25 p‐EMT‐related genes in Roessler's data of HCC.^19^ Difference of mRNA expression was compared by Students' *t* test. Cut‐off of *P* value and fold change were as following: *P* value: .01, fold change: 1.5.

### 2.5 Kaplan‐Meier Plotter analysis

The correlation between mRNA expression levels of p‐EMT‐related genes and prognosis of HCC patients was evaluated using Kaplan‐Meier Plotter (www.kmplot.com), an online database containing information about the effects of 54 675 genes on survival in more than 20 types of cancers.^20^ Each p‐EMT‐related gene was first entered into ‘Liver cancer’ item in this database. HCC patients were divided into high and low expression group according to median values of mRNA expression and then Kaplan‐Meier overall survival curves were generated. Significant difference was considered when logrank *P* value <.05.

### 2.6 Functional enrichment analysis

Database for Annotation, Visualization and Integrated Discovery (https://david.ncifcrf.gov/)^21^ was utilized to perform Gene Ontology (GO) functional annotation and Kyoto Encyclopedia of Genes and Genomes (KEGG) pathway enrichment analysis of the 25 p‐EMT‐related genes. Information about the enriched GO terms and KEGG pathways with *P* < .05 was downloaded from the webpage and visualized using Python software.

### 2.7 The cBioPortal database analysis

The cBioPortal (www.cbioportal.org) is an online open website resource capable to assess multidimensional cancer genomics data.^22^ In this study, we analysed the genomic profiles of the 25 p‐EMT‐related genes, which contained mutations, putative copy‐number alterations from GISTIC and mRNA Expression *z*‐Scores (RNASeq V2 RSEM) with a z‐score threshold ± 1.8. The correlations of p‐EMT‐related genes with each other were analysed via the cBioPortal online tool and visualized by using ggcorplot package of R software. Pearson's correction was included.

### 2.8 Protein‐protein interaction (PPI) network

The PPI interaction network between the p‐EMT‐related genes was constructed by Search Tool for the Retrieval of Interacting Genes (STRING) database (http://string‐db.org/)^23^ and visualized by Cytoscape software, an open source platform for visualizing complex networks.^24^

### 2.9 Prediction of miRNAs and lncRNAs

We predicted the upstream miRNAs of the six key p‐EMT‐related genes by utilizing miRTarbase (http://mirtarbase.mbc.nctu.edu.tw/php/index.php), an online database containing more than thousands of miRNA‐target interactions validated experimentally by reporter assay, Western blot, microarray and next‐generation sequencing experiments.^25^ For the credibility of predicted results, only the miRNA‐target interactions validated by reporter assay were selected for further analysis. The upstream candidate lncRNAs interacted with key miRNAs were predicted by using the miRNet database (https://www.mirnet.ca/), an online platform providing comprehensive analyses of miRNA‐ target interactions. Selection criteria were ‘Organism‐H.sapies’ and ‘target type‐lncRNAs’. The expression levels and prognostic values of predicted miRNAs and lncRNAs were analysed between HCC tissues and normal tissues using starBase v3.0 (http://starbase.sysu.edu.cn/index.php), an online database providing information on differential expression, survival and coexpression analysis of RNAs data from the TCGA projects.^26^ *P* value <.05 was considered as statistically significant. We drew the Venn diagram using VENNY 2.1.0 (http://bioinfogp.cnb.csic.es/tools/venny/index.html), an interactive online tool.

### 2.10 Correlation analysis

We explored the correlations of mRNA‐miRNA, miRNA‐lncRNA and mRNA‐lncRNA pairs in HCC using starBase v3.0 database and *P* value <.05 was considered as statistically significant.

## 3 RESULTS

### 3.1 Complete EMT may not be necessary for HCC metastasis

E‐cadherin has been considered as a hall mark of EMT.^3^ Firstly, we performed a comprehensive analysis of E‐cadherin as a marker for complete EMT in HCC. We evaluated the expression patterns of E‐cadherin across multiple cancer types in TCGA and the differential expression level of E‐cadherin in HCC was not very obvious compared with many other cancers (Figure 1A). Analysis of 12 HCC cohorts in the HCCDB database revealed that mRNA expression level of E‐cadherin was lower in HCC tissues than in adjacent nontumour tissues in 8 of 12 cohorts (Figure 1B), and no difference of E‐cadherin expression was observed in the other cohorts. Intriguingly, prognostic values of E‐cadherin for HCC were not significant in all of GEO, TCGA and ICGC‐LIRI‐JP datasets (Figure 1C). IHC staining showed that E‐cadherin was mainly localized in the cell membrane. There was no significant difference between the protein expression level of E‐cadherin in HCC tissues and adjacent nontumour tissues (Figure 1D). Moreover, E‐cadherin expression might have no correlations with clinical features of HCC, including individual cancer stages, tumour grade and nodal metastasis status (Figure S1). Considering the results above, we thought the complete EMT might not be necessary for HCC progression and concentrated our attention on the role of p‐EMT‐related genes in HCC.

### 3.2 Different mRNA expression levels of p‐EMT‐related genes in HCC patients

**FIGURE 2:**
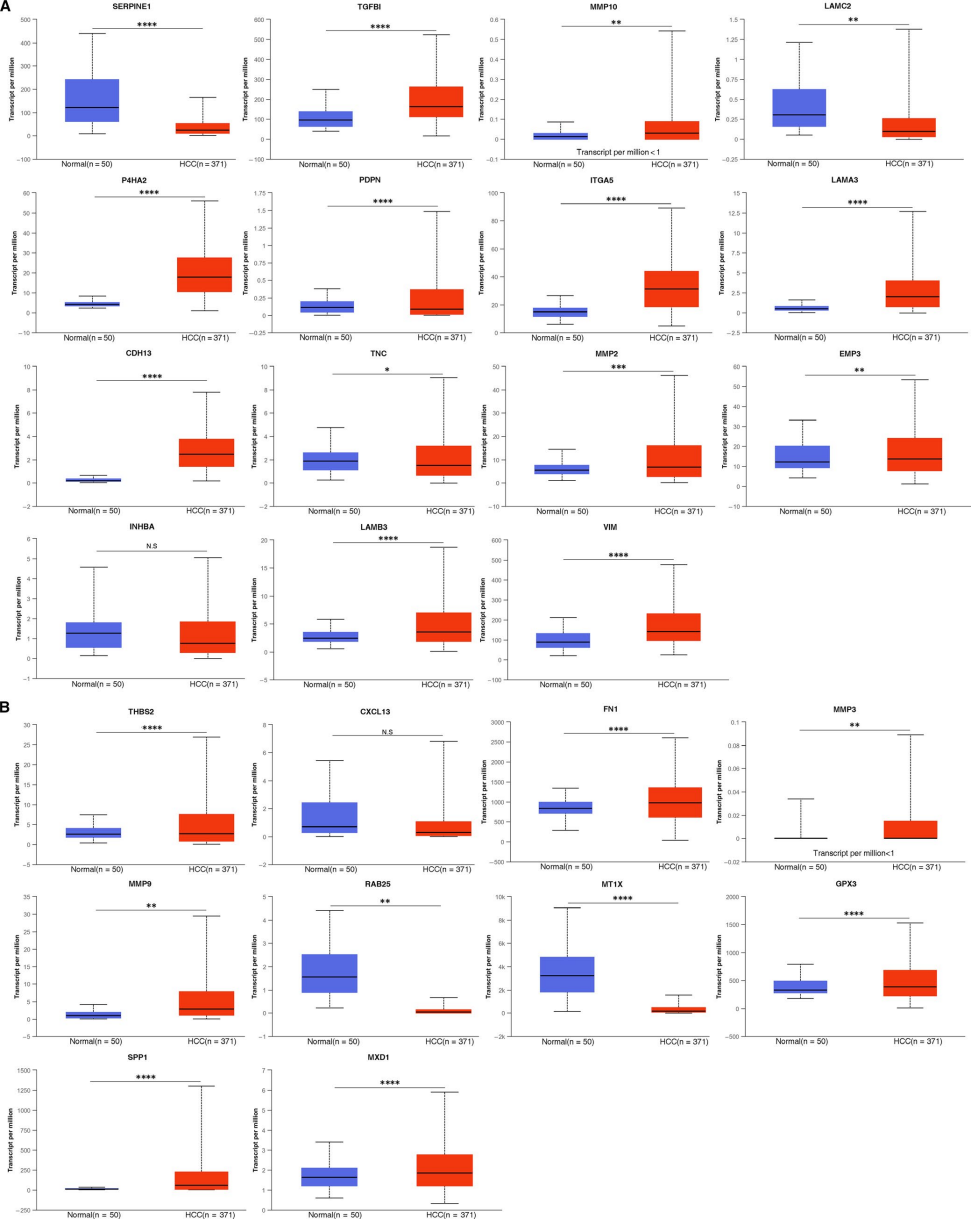
The mRNA expression of 25 p‐EMT‐related genes in HCC. The mRNA expression levels of 25 p‐EMT‐related genes in HCC

We used UALCAN to compare the mRNA expression of p‐EMT‐related genes in HCC tissues and normal liver tissues (Figure 2 and Figure S2). The expression patterns of these genes were further confirmed the results in Oncomine and GEPIA (Figures S3 and S34). Among common p‐EMT‐related genes, the mRNA expression levels of P4HA2, ITGA5, LAMA3, CDH13, LAMB3, VIM were found significantly higher in HCC tissues than in normal tissues in all databases. Up‐regulation of variable p‐EMT‐related genes MMP9 and SPP1 was observed in HCC tissues, while MT1X was remarkably down‐regulated in HCC tissues.

### 3.3 Correlation between the mRNA expression levels of p‐EMT‐related genes and prognosis of HCC patients

**FIGURE 3:**
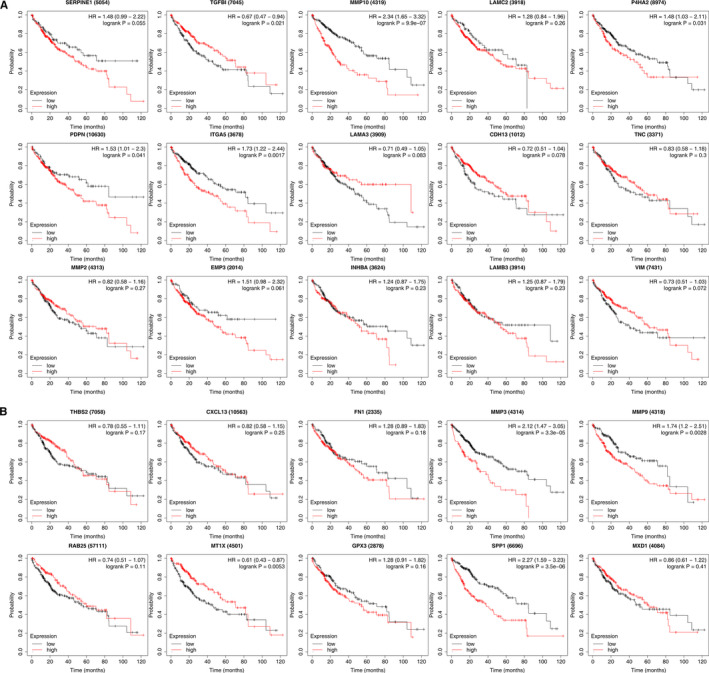
Prognostic value of mRNA expression levels of distinct p‐EMT‐related genes in HCC patients. The correlation between mRNA expression levels of distinct p‐EMT‐related genes and overall survival of HCC patients

To determine the role of p‐EMT‐related genes in prognosis of HCC patients, we analysed the correlation between the mRNA expression levels of p‐EMT‐related genes and the survival of patients with HCC by using Kaplan‐Meier plotter. The results showed that the increased mRNA expression levels of MMP10, P4HA2, PDPN, ITGA5, MMP3, MMP9 and SPP1 were significantly associated with worse overall survival of HCC patients (*P* < .05). However, HCC patients with higher mRNA expression levels of TGFBI and MT1X showed better prognosis (Figure 3).

We selected common p‐EMT‐related genes P4HA2, ITGA5 and variable p‐EMT‐related genes MMP9, MT1X, SPP1 as key genes according to the criterion of converse expression pattern and survival prognosis. The mRNA expression levels of them all were remarkably correlated with patients’ individual cancer stages (Figure S4).

### 3.4 Functional enrichment analysis for p‐EMT‐related genes

**FIGURE 4:**
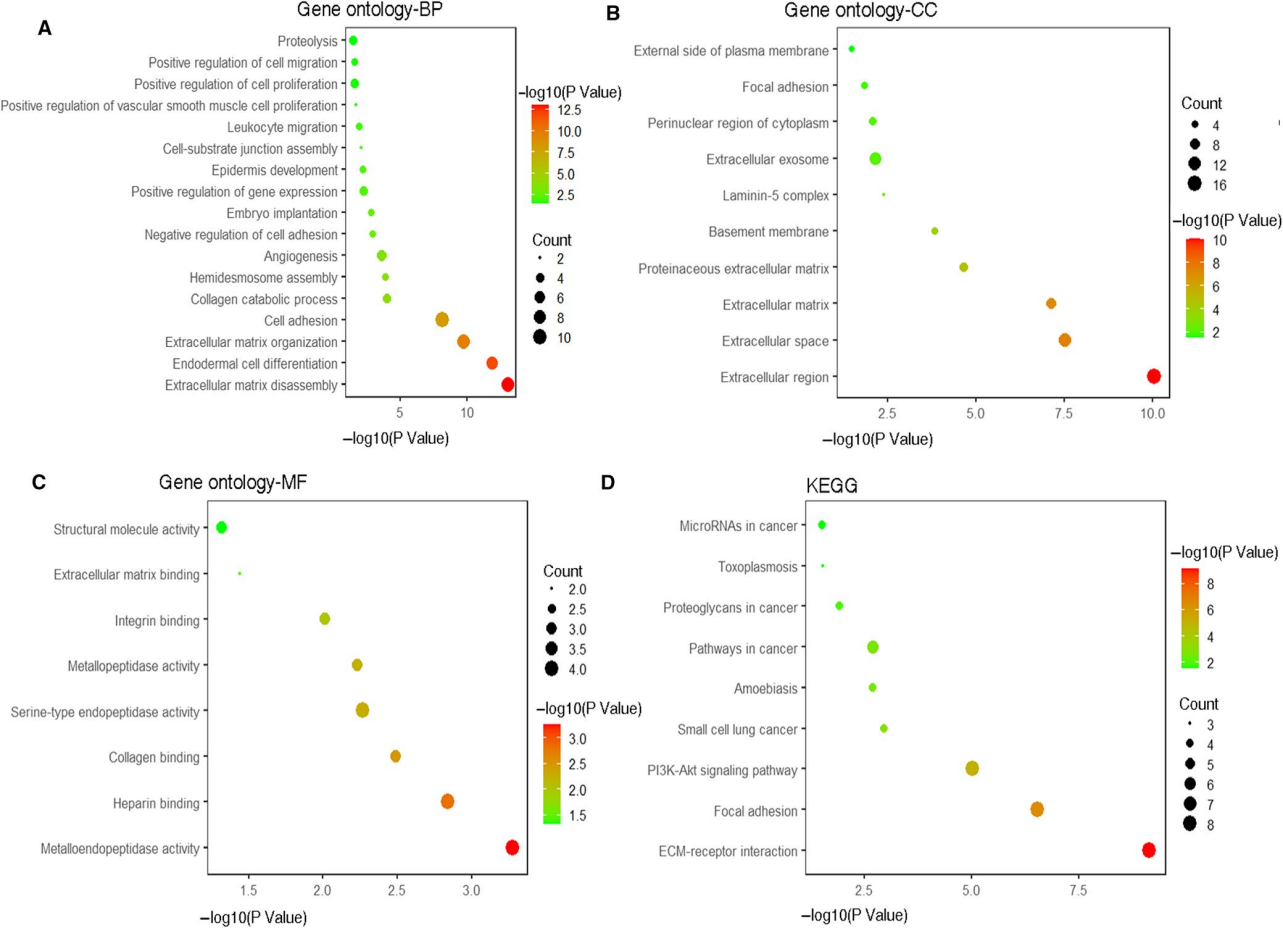
Functional enrichment analysis of 25 p‐EMT‐related genes. A, Gene ontology analysis of biological process for 25 p‐EMT‐related genes. B, Gene ontology analysis of cellular component for 25 p‐EMT‐related genes. C, Gene ontology analysis of molecular function for 25 p‐EMT‐related genes. D, KEGG analysis of p‐EMT‐related genes

In addition to biological processes identified already, the p‐EMT programme may be associated with some other biological processes and pathways. We performed functional enrichment analysis to explore the particular biological functions of the 25 p‐EMT related genes. Consistent with previous studies, the results showed that these genes were mainly enriched in extracellular matrix disassembly and organization. Moreover, KEGG pathways including ECM‐receptor interaction, focal adhesion were closely related to p‐EMT‐related genes. Besides the common functions, these genes were also found to have close relationship with endodermal cell differentiation, leucocyte migration, PI3K‐Akt signalling pathway, and several pathways in cancer, which indicated the implication of 25 p‐EMT‐related genes in cancer (Figure 4A‐D).

### 3.5 Genetic mutations in p‐EMT‐related genes and PPI network construction

**FIGURE 5:**
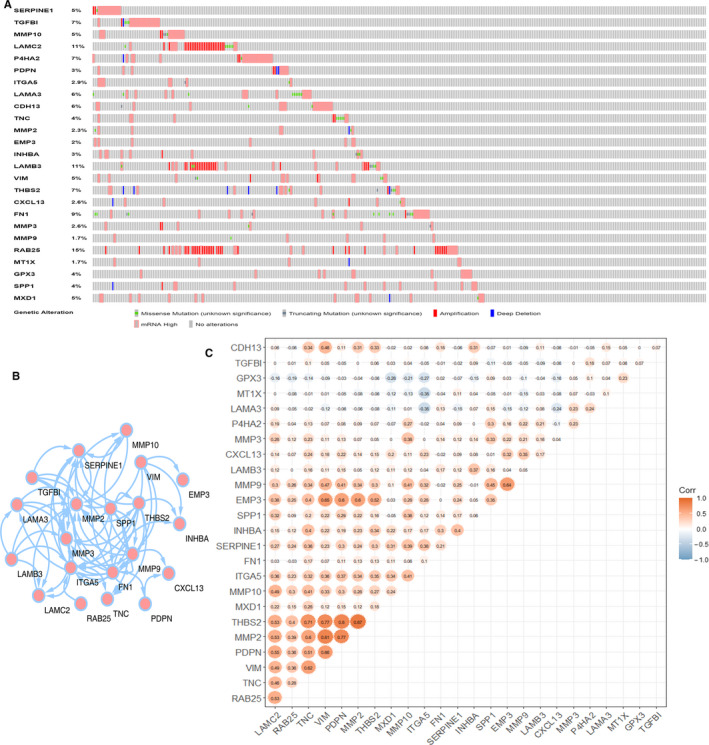
Analysis of genetic mutations, mutual interaction and correlations with each other in 25 p‐EMT‐related genes. A, Genetic mutations analysis of 25 p‐EMT‐related genes in HCC. B, The PPI network for 25 p‐EMT‐related genes. C, Corrections of different p‐EMT‐related genes with each other in HCC

The alterations of p‐EMT‐related genes were explored by using cBioPortal online tool for liver hepatocellular carcinoma (TCGA, PanCancer Atlas). The 25 p‐EMT‐related genes were altered in 222 samples of 348 patients with HCC (64%). More than two types of alterations of these genes were detected in HCC samples including mutation, amplification, depletion and mRNA alterations (Figure 5A). To figure out the mutual interactions of p‐EMT‐related genes, we utilized Cytoscape and String to construct a PPI network (Figure 5B). The result showed that 20 of p‐EMT‐related genes might have interactions with others. In addition, correlations of different p‐EMT‐related genes expressions with each other were showed in the correlation heatmap and Pearson's correction was included (Figure 5C). The result demonstrated a low to high correlation among several p‐EMT‐related genes. For example, THBS2 expression was observed to be closely correlated with MMP2, while there was no correlation between P4HA2 and INHBA expression.

### 3.6 Identification of key upstream miRNAs

**FIGURE 6:**
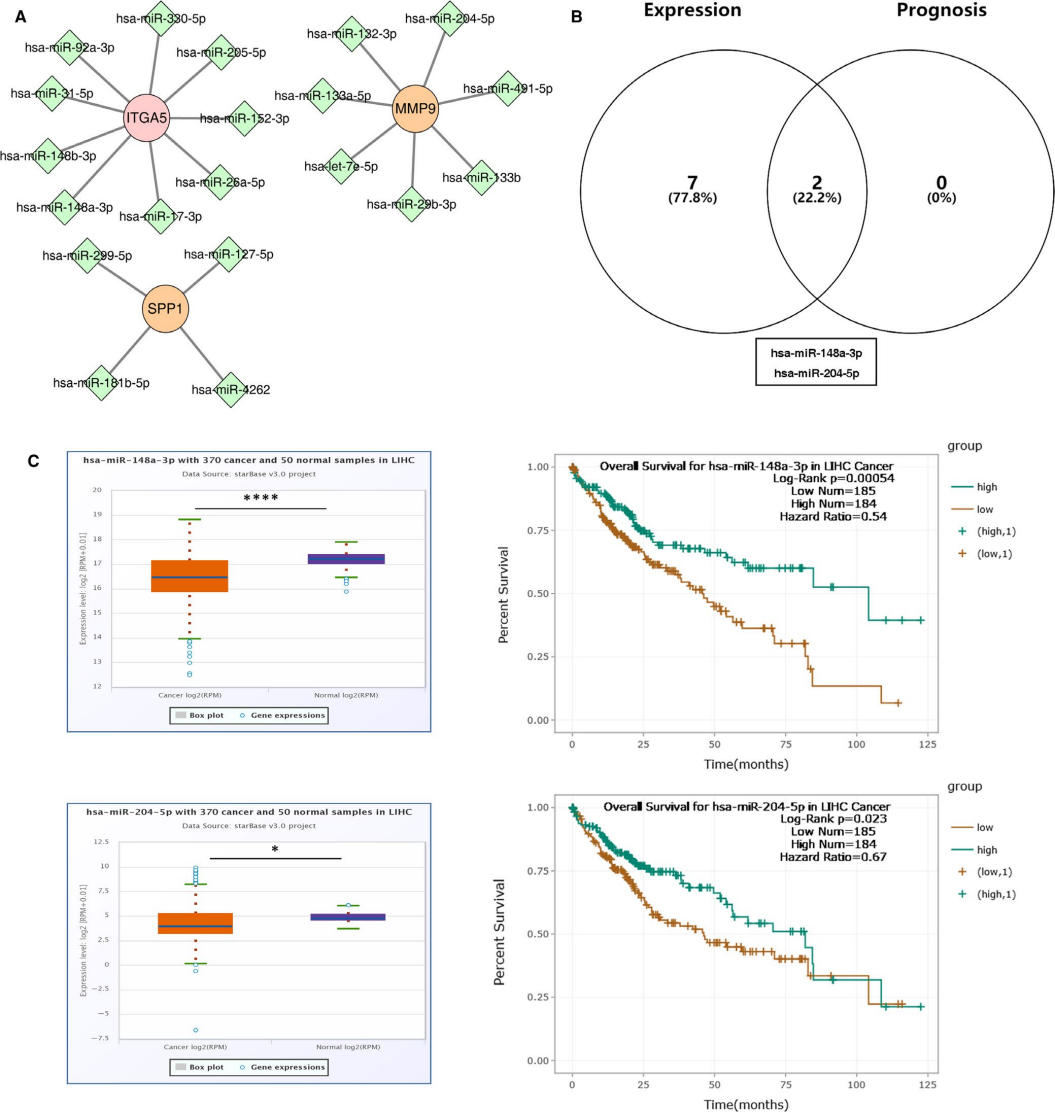
Prediction of key upstream miRNAs. A, The mRNA‐miRNA network containing selected key p‐EMT‐related genes. B, Identification of the key upstream miRNAs with low expression and poor prognosis values in the predicted candidate miRNAs. C, The expression and survival analysis of miR‐148a‐3p and miR‐204‐5p in HCC

Then, we sought to establish an mRNA‐miRNA‐lncRNA competing endogenous RNA subnetwork associated with p‐EMT‐related genes in HCC, among which each component was markedly correlated with HCC prognosis. In order to screen key miRNAs regulating these five key p‐EMT‐related genes, we predicted upstream miRNA of the five key genes by using miRTarBase, an experimentally validated miRNA‐target interactions database. To improve the credibility of the predicted results, only miRNA‐target interactions validated by reporter assays were included in our study. A total of 20 miRNAs were predicted to regulate the three key p‐EMT‐related genes (ITGA5, MMP9 and SPP1), all of which were up‐regulated in HCC tissues (Figure 6A). There was no miRNA noticed to target P4HA2 and MT1X in this database. Given the inverse regulatory relationship between miRNAs and their target genes, we explored the expression and prognostic values of the predicted miRNA in HCC patients by using starBase v3.0. The result showed that seven miRNAs were significantly down‐regulated in HCC tissues, and only two of them (has‐miR‐148a‐3p and has‐miR‐204‐5p) were associated with poor prognosis in HCC patients (Figure 6B). The expression boxplot and survival curve of the two candidate miRNAs were shown in Figure 6C, and they were selected as key miRNAs for next analysis.

### 3.7 Identification of key upstream lncRNAs

**FIGURE 7:**
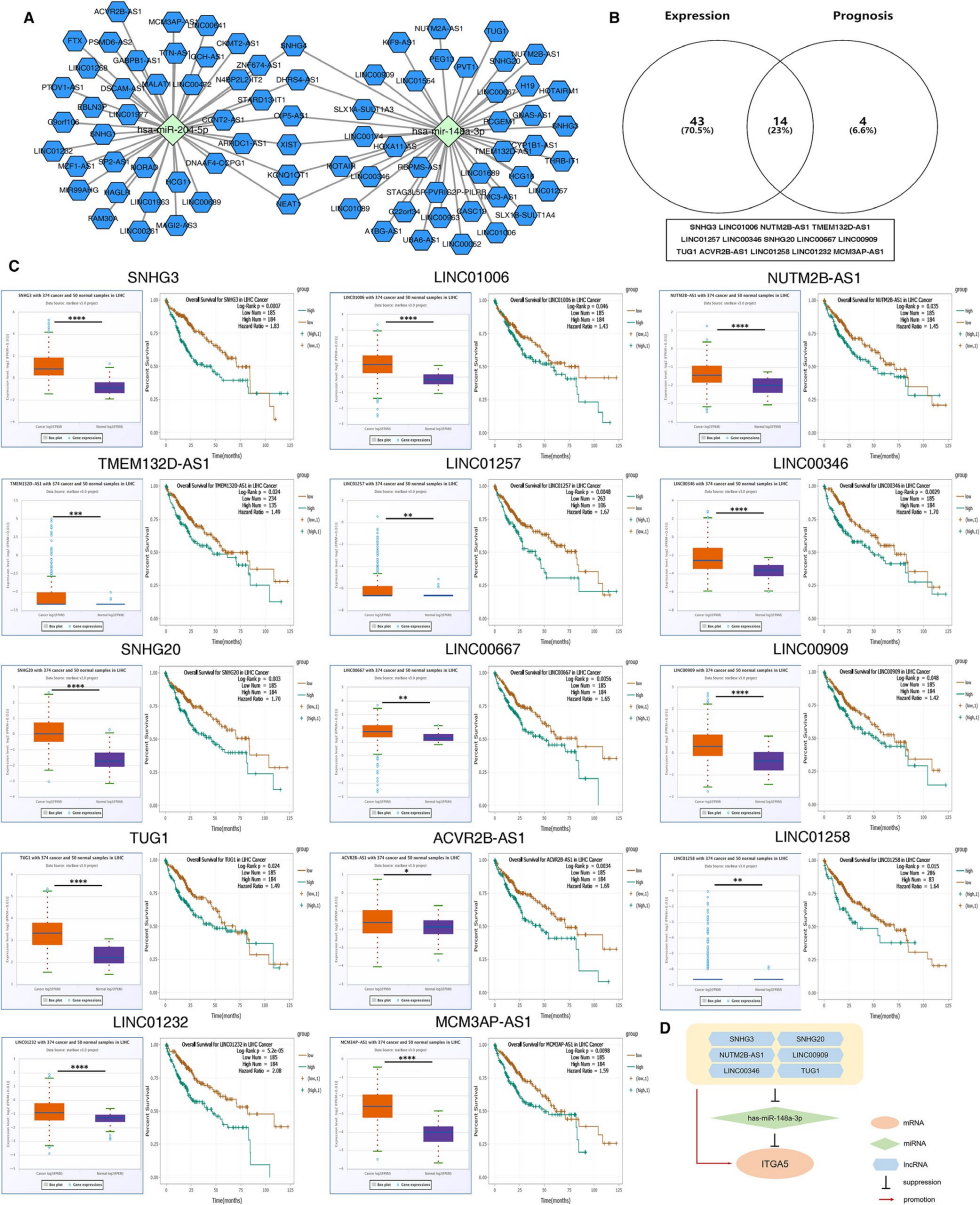
Prediction of key upstream lncRNAs. A, The miRNA‐lncRNA network containing selected key upstream miRNAs. B, Identification of the key upstream lncRNAs with high expression and poor prognosis values in the predicted candidate lncRNAs. C, The expression and survival analysis of 14 selected candidate lncRNAs in HCC. D, The potential mRNA‐miRNA‐lncRNA regulatory subnetwork associated with prognosis of HCC

It is well known that lncRNA can act as sponge to competitively bind to miRNA, regulating expression of target genes. Hence, we predicted key upstream lncRNAs that potentially bound to the two key miRNAs by miRNet, an online database for miRNA‐associated studies. A total of 80 lncRNAs were discovered in the database for the two miRNAs has‐miR‐148a‐3p and has‐miR‐204‐5p (Figure 7A). The ceRNA hypothesis believes that lncRNA could weaken miRNA activity to up‐regulate expression of miRNA‐related target genes.^13^ Based on the hypothesis, there should be a negative correlation between lncRNA and miRNA. Thus, we analysed the expression patterns and survival curves of these lncRNAs in HCC using starBase v3.0. Compared with in normal tissues, only 57 out of 80 lncRNAs were dramatically up‐regulated in HCC tissues. Survival analysis showed that patients with high expression of 18 lncRNAs had poor prognosis (Figure 6B). Combining expression and survival analysis results, 14 lncRNAs were screened as candidate lncRNAs for the ceRNA network (Figure 7B‐C).

### 3.8 Construction of the mRNA‐miRNA‐lncRNA subnetwork associated with prognosis in HCC

According to the ceRNA hypothesis, the key eligible lncRNAs in ceRNA subnetwork should be negatively correlated with miRNA and meanwhile positively correlated with mRNA. Thus, we analysed the correlation between the key p‐EMT‐related genes mRNA and candidate miRNAs, miRNAs and candidate lncRNAs as well as mRNA and lncRNAs. As a result, one of two mRNA‐miRNA pair (ITGA5‐ miR‐148a‐3p), six of 14 miRNA‐lncRNA pairs (miR‐148a‐3p‐SNHG3, miR‐148a‐3p‐NUTM2B‐AS1, miR‐148a‐3p‐LINC00346, miR‐148a‐3p‐SNHG20, miR‐148a‐3p‐LINC00909 and miR‐148a‐3p‐TUG1) and six of 14 mRNA‐lncRNA pairs (ITGA5‐SNHG3, ITGA5‐NUTM2B‐AS1, ITGA5‐LINC00346, ITGA5‐SNHG20, ITGA5‐LINC00909 and ITGA5‐TUG1) were fitted with the ceRNA mechanism (Supplementary Table S1 and Figures S5 and S6). Taken these three levels into consideration, we constructed an mRNA‐miRNA‐lncRNA subnetwork correlated with p‐EMT‐related genes, and each component of which had associations with prognosis of HCC (Figure 7D). The subnetwork might be developed as promising diagnostic biomarkers or therapeutic targets concerning p‐EMT programme for HCC.

### 3.9 Identification of the protein expression levels of key p‐EMT‐related genes in HCC

**FIGURE 8:**
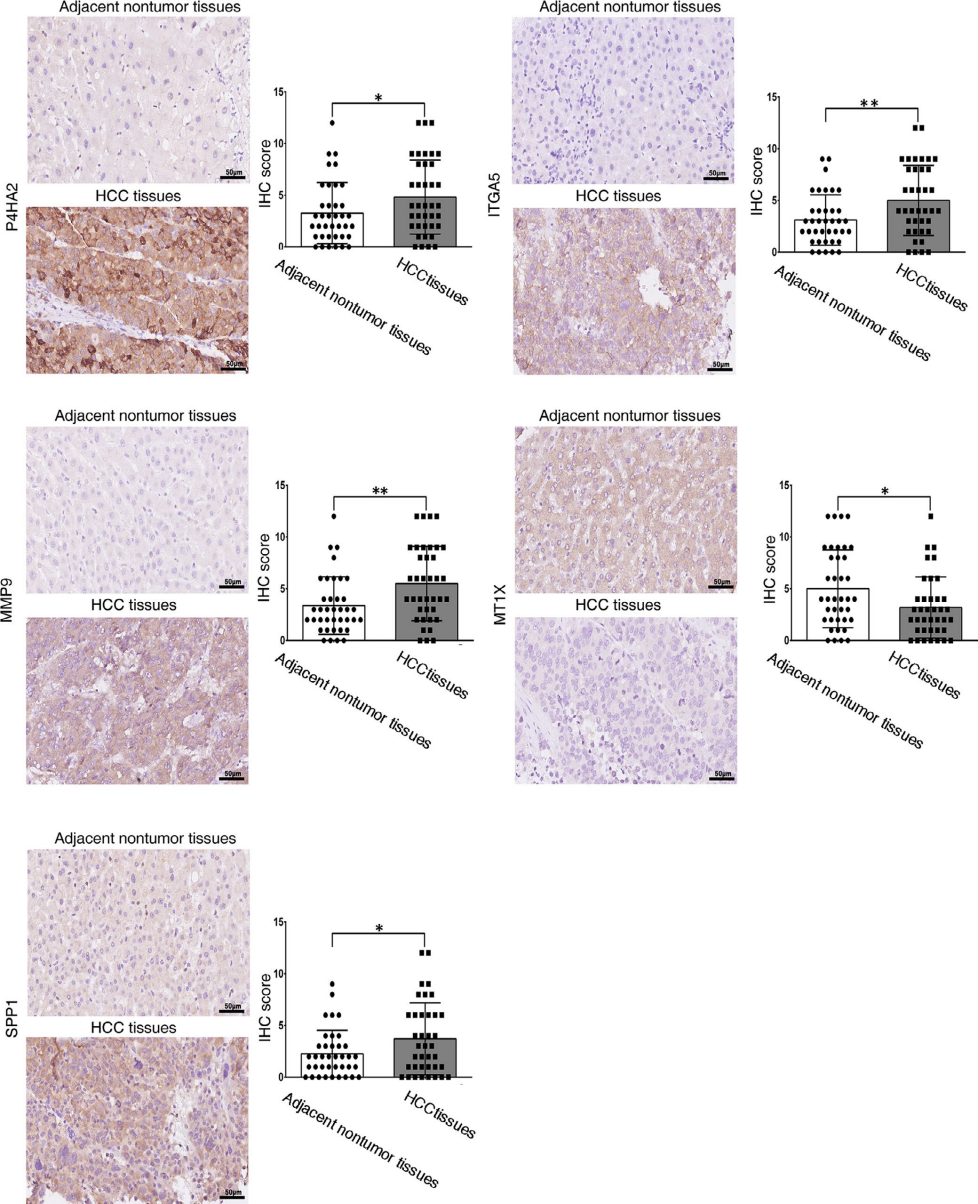
IHC assays of P4HA2, ITGA5, MMP9, MT1X and SPP1 in HCC tissues and adjacent nontumour tissues

In addition to mRNA expression analysis, we further observed the protein expression levels of the five key p‐EMT‐related genes in HCC tissues and corresponding adjacent nontumour tissues using IHC assay. The results showed that most of the five key genes were mainly localized in the extracellular region, cytoplasm and cell membranes. Consistent with previous results, the protein expression levels of P4HA2, ITGA5, MMP9 and SPP1 were higher in HCC tissues than in adjacent nontumour tissues, while MT1X showed the opposite results in protein expression level. Representative IHC images and IHC score of the five key p‐EMT‐related genes were presented in Figure 8.

## 4 DISCUSSION

Increasing evidence has identified the vital role of EMT in cancer progression, in which E‐cadherin dysregulation was observed in many types of cancers,^6^, ^27^ including HCC. In our study, we found that E‐cadherin expression was lower in HCC tissues in few cohorts and no significant difference was observed in the others. Analysis of prognostic values of E‐cadherin and its association with clinical features in HCC showed that E‐cadherin may be not necessary for malignancy of HCC, indicating the EMT model may not be the most suitable for HCC progression.

During HCC progression, EMT represents the first step of HCC metastasis, where epithelial cells in the primary tumour lose cell‐cell adhesion and gain the capacity of migration and invasion into blood vessels. Upon intravasation, these cells remain in the bloodstream until they colonize distant organs to seed micrometastasis. Then, these cells undergo a reversal of EMT‐MET to restore their epithelial features and form secondary tumours, thereby completing the cascade of metastasis and invasion.^28^ The intermediate p‐EMT programme in the process has achieved more and more attention because of its importance. Cancer cells undergoing p‐EMT state function like cancer cells with mesenchymal state, yet they do not lose epithelial characteristics completely.^29^ Recently, it has been recognized that the ability of cancer cells with p‐EMT state, rather than complete EMT, possesses higher metastatic risks.^30^ In addition, p‐EMT programme may be linked to functions of cancer stem cells and progenitor cell, resulting in multiple cell phenotypes through self‐renewal or asymmetric divisions.^31^ During this process of p‐EMT, multiple relevant genes altered in HCC, which was implicated with HCC progression. Although several p‐EMT‐related genes have been confirmed to be potential targets of HCC, the distinct roles of the identified 25 p‐EMT‐related genes in HCC and regulatory ceRNA networks remain to be elucidated. This study is the first time to explore the expression, prognostic values, functional enrichment analysis of 25 p‐EMT‐related genes in HCC and identify five key p‐EMT‐related genes according to the criterion of converse expression and survival. Moreover, an mRNA‐miRNA‐lncRNA ceRNA network regulating the key p‐EMT‐related genes correlated with prognosis of HCC was also constructed. The workflow was shown in Figure S7. We hope that our findings will contribute to available knowledge about improvement of therapy strategy and prognosis accuracy for patients with HCC.

In this study, we explored the expression patterns and prognosis values of 25 p‐EMT‐related genes and identified five key p‐EMT‐related genes associated with prognosis of HCC, including two common p‐EMT‐related genes P4HA2, ITGA5 and three variable p‐EMT‐related genes MMP9, MT1X, SPP1. Genetic mutation analysis showed that amplification was the most common type of genetic mutations in HCC patients and the number of patients with RAB25 mutation was the highest. PPI network and correlation analysis of the 25 p‐EMT‐related genes suggested that most of these genes had close relationships with each other. Functional enrichment analysis revealed that the 25 p‐EMT‐related genes are enriched in malignant behaviours associated with cancers, including angiogenesis, cell adhesion, positive regulation of cell migration and proliferation. Indeed, previous studies have reported the involvement of these genes in malignancy. P4HA2, an essential enzyme during collagen formation, has been reported to be an indicator of many types of cancers progression. In vitro and in vivo experiments are used to clarify the mechanisms of aspirin in the suppression of HCC targeting abnormal collagen deposition associated with P4HA2^32^ and HBx is capable to promote hepatocarcinogenesis through miR‐30e targeting P4HA2 mRNA.^33^ ITGA5 belongs to the integrin alpha chain family and interacts with the beta 1 subunit to form a fibronectin receptor.^34^ ITGA5 can be considered as a mesenchymal marker, overexpression of which is correlated with HCC proliferation, differentiation, invasion and metastasis.^35^, ^36^ MMP9 has been considered as biomarker for prognosis of many types of solid tumours, including HCC.^37^ Transforming growth factor (TGF)‐β signalling plays an important role at the early stage of EMT programme in HCC development and MMP9 is a key target of the tumour‐progressive TGF‐β signalling, which might be a potential target for HCC therapy.^38^ The protein encoded by SPP1 can be secreted into serum and is related with the attachment of osteoclasts to mineralized bone matrix.^39^ SPP1 has been screened as a molecule for HCC diagnosis and prognosis.^40^ Our results showed that the four p‐EMT‐related genes P4HA2, ITGA5, MMP9 and SPP1 were overexpressed in HCC tissues compared with in normal liver tissues, and the mRNA expression levels of them all were associated with prognosis of patients with HCC. However, MT1X was identified as a tumour suppressor involved in HCC progression and metastasis.^41^ The expression and survival analysis showed that MT1X mRNA expression was higher in normal tissues and correlated with better prognosis of HCC patients.

Considering the importance of ceRNA networks in cancer, we constructed a novel ceRNA subnetwork associated with prognosis of HCC, containing the key p‐EMT‐related genes we selected, to explore the regulatory mechanisms of p‐EMT‐related genes. Therefore, candidate miRNAs and lncRNAs binding to potential miRNAs were subsequently predicted. 20 miRNAs of ITGA5, MMP9 and SPP1 were first identified using miRTarBase database. Given the action mechanism of miRNA on mRNA, we identified the ITGA5‐miR‐148a‐3p pair for further analysis by performing correlation analysis for these mRNA‐miRNA interactions in HCC using starBase v3.0. Low expression of miR‐148a‐3p was an independent risk factor for overall survival for HCC.^42^ In vitro experiment showed that miR‐148a‐3p inhibited the growth of HCV‐infected HCC cells by targeting c‐Jun mRNA.^43^ Then, lncRNAs binding to miR‐148a‐3p were predicted by miRNet databases. Based on the ceRNA hypothesis, we predicted six upstream lncRNAs (SNHG3, NUTM2B‐AS1, LINC00346, SNHG20, LINC00909 and TUG1), which were remarkably up‐regulated in HCC and associated with poor prognosis of HCC. Most of these lncRNAs have been reported the oncogenic roles of lncRNAs in HCC. For example, SNHG3 promotes EMT and sorafenib resistance via activating miR‐128/CD151 axis in HCC.^44^ LINC00346 promotes HCC development via JAK‐STAT3 signalling pathway activation.^45^ Overexpression of SNHG20 has been reported to be an indicator of poor prognosis of HCC.^46^ In addition, TUG1 is overexpressed in HCC and enhances cell proliferation and tumorigenicity by epigenetically inhibition of Kruppel‐like factor 2 (KLF2) transcription.^47^ In consequence, a novel mRNA‐miRNA‐lncRNA regulatory subnetwork associated with prognosis of HCC was constructed successfully. Interestingly, we found that some interactions in this network were identified in previous studies, which further confirmed the reliability of our results.^48^ In the end, six pairs of subnetwork (SNHG3/SNHG20/NUTM2B‐AS1/LINC00909/LINC00346/TUG1‐miR‐148a‐3p‐ITGA5) were acceptable, which might be utilized to be prognostic biomarkers for HCC.

We further identified the protein expression levels of the five key p‐EMT‐related genes in HCC tissues and corresponding adjacent nontumour tissues using IHC staining. Consistent with results above, the expression of P4HA2, ITGA5, MMP9 and SPP1 was higher in HCC tissues than in adjacent nontumour tissues in protein expression level. Nevertheless, the protein expression level of MT1X was lower in HCC tissues compared with nontumour liver tissues.

In conclusion, we systematically analysed the expression patterns, prognostic values, genetic mutations of p‐EMT‐related genes as well as their correlation with each other in HCC, and identified five key p‐EMT‐related genes in HCC. Moreover, a novel mRNA‐miRNA‐lncRNA ceRNA subnetwork containing p‐EMT‐related genes associated with HCC was constructed, each component of which possessed high prognostic value for HCC. More in vitro and in vivo experiments should be conducted to confirm the role of the subnetwork and p‐EMT programme in HCC. Our findings suggested potential therapeutic and prognostic values of p‐EMT‐related genes as well as their ceRNA regulatory subnetwork for the improvement of survival and prognostic accuracy in HCC.

## CONFLICT OF INTEREST

The authors report no conflicts of interest in this work.

## AUTHOR CONTRIBUTION

**Yu Lei:** Investigation (equal); Methodology (equal); Visualization (equal); Writing‐original draft (equal). **Wei Yan:** Funding acquisition (supporting); Visualization (supporting). **Zhuoying Lin:** Methodology (supporting). **Jingmei Liu:** Methodology (supporting). **De‐An Tian:** Funding acquisition (supporting); Visualization (lead). **Han Ping:** Funding acquisition (lead); Writing‐original draft (lead); Writing‐review & editing (lead).

## ETHICS AND CONSENT APPROVAL

This study was approved by the Academic Committee of Tongji Medical College, Huazhong University of Science and Technology. Written informed consent from the patients was obtained.

## Supporting information

Fig S1

Fig S1

Fig S3

Fig S4

Fig S5

Fig S6

Fig S7

Fig S8

Fig S9

Fig S10

Fig S11

Fig S12

Fig S13

Fig S14

Fig S15

Fig S16

fig S17

Fig S18

Fig S19

Fig S20

Fig S21

Fig S22

Fig S23

Fig S24

Fig S25

Fig S26

Fig S27

Fig S28

Fig S29

Fig S30

Fig S31

Fig S32

Fig S33

Fig S34

Table S1

## Data Availability

All the data in our study can be accessed from the online database.
