# Supplementary figures and images for: Comprehensive analysis of partial epithelial mesenchymal transition‐related genes in hepatocellular carcinoma
Source: J Cell Mol Med. 2020 Nov 20;25(1):448–62. doi: 10.1111/jcmm.16099 (PMC7810929; doi:10.1111/jcmm.16099)

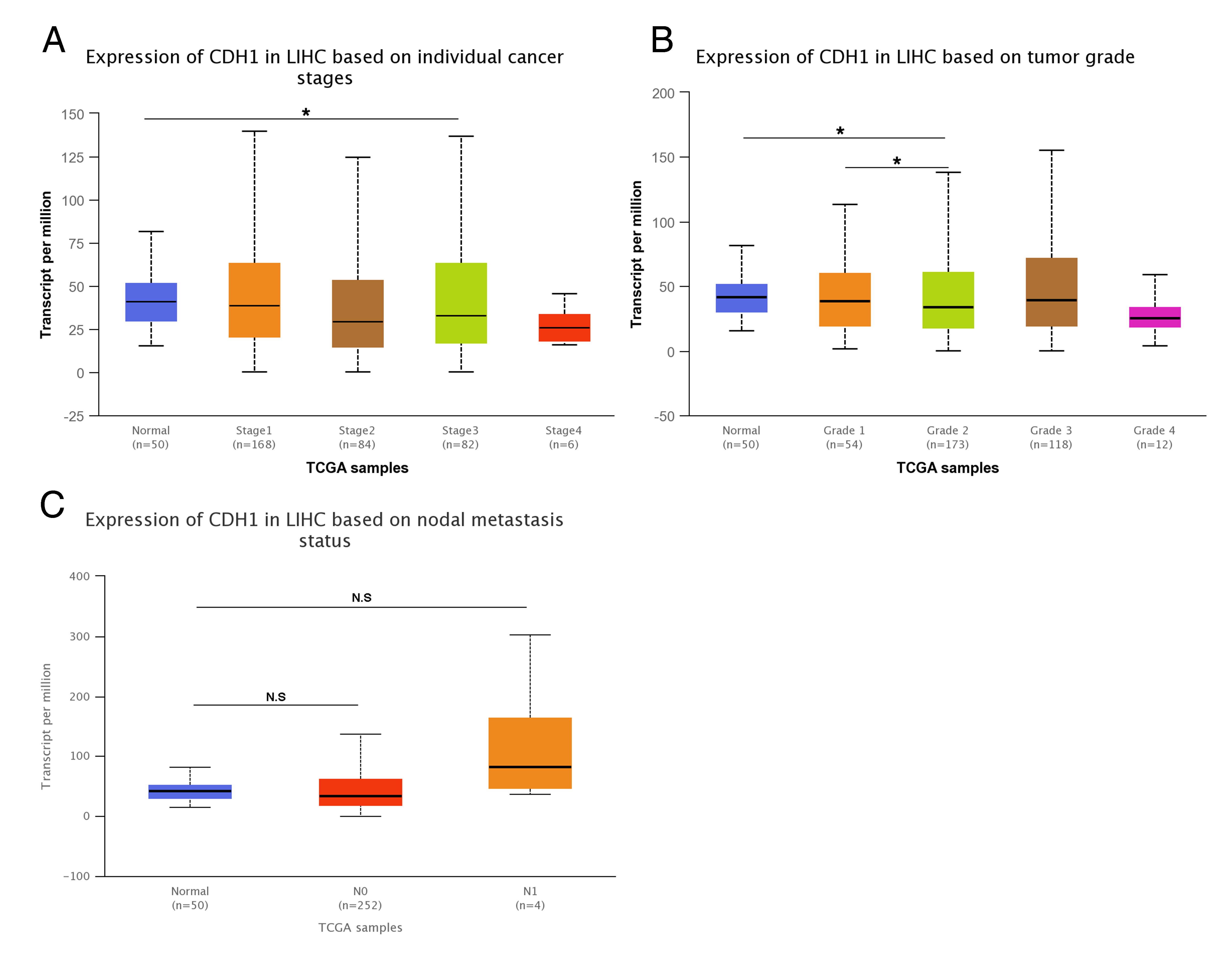

Supplement: Supplementary file 1 — Fig S1 [file JCMM-25-448-s001.tif]

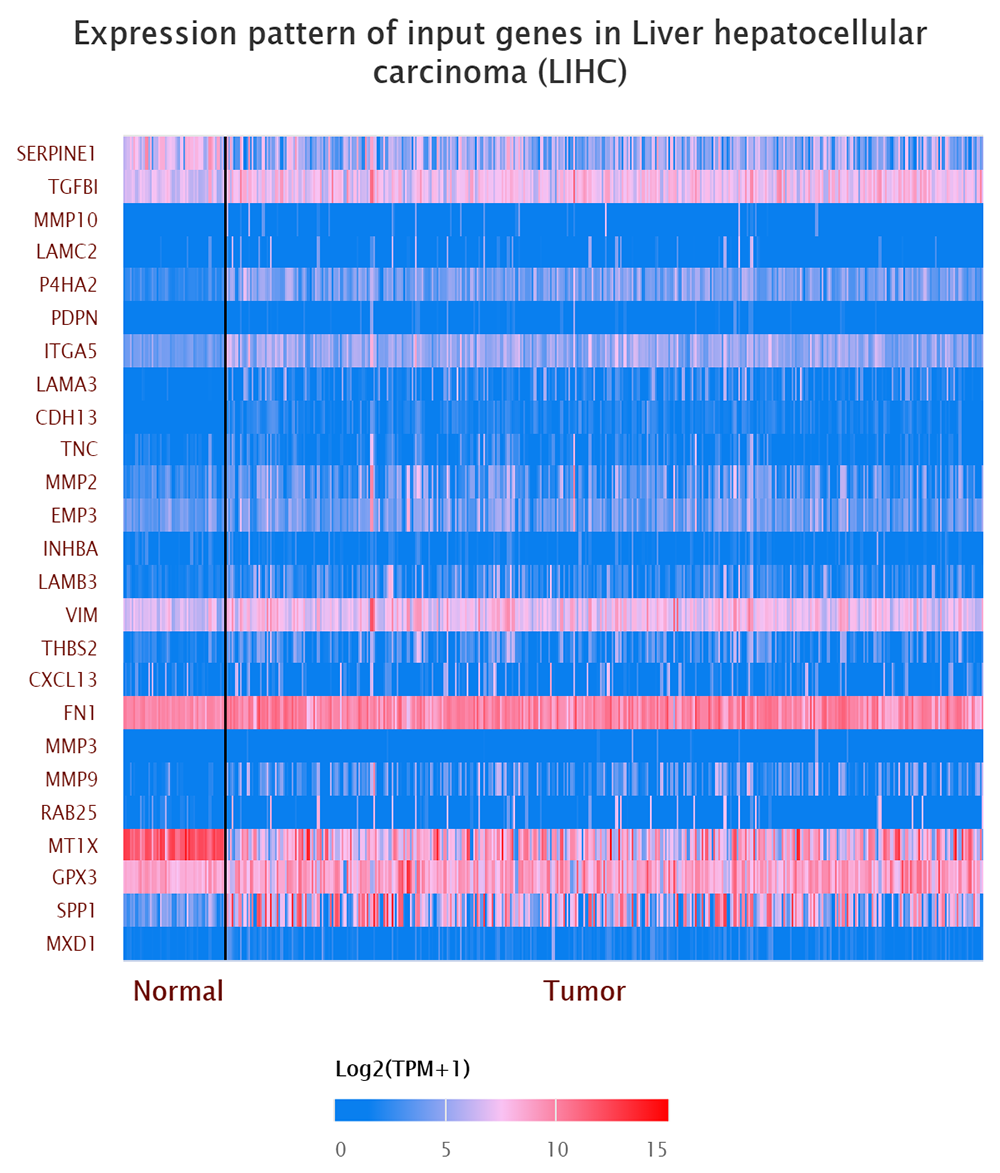

Supplement: Supplementary file 2 — Fig S1 [file JCMM-25-448-s002.tif]

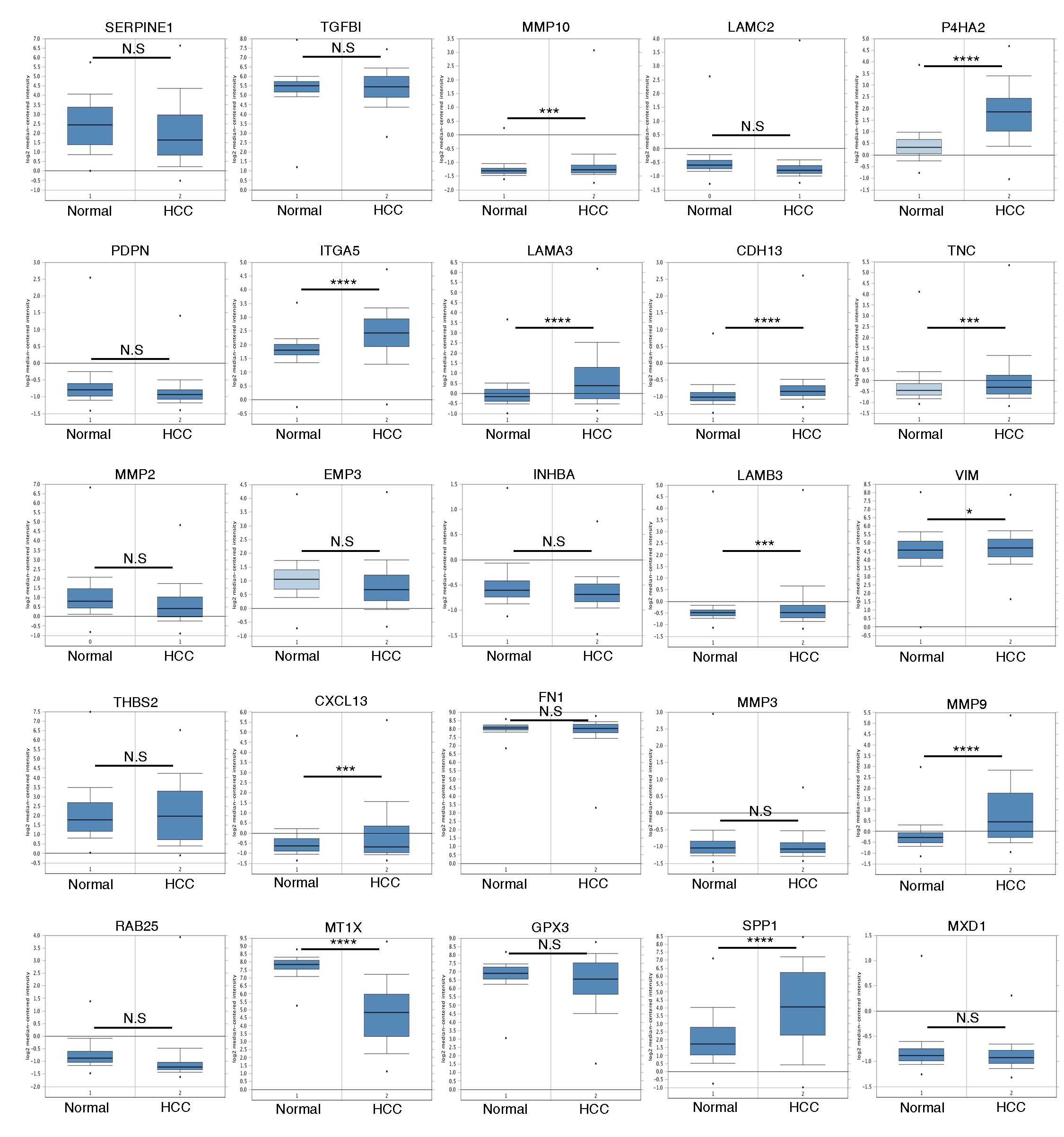

Supplement: Supplementary file 3 — Fig S3 [file JCMM-25-448-s003.tif]

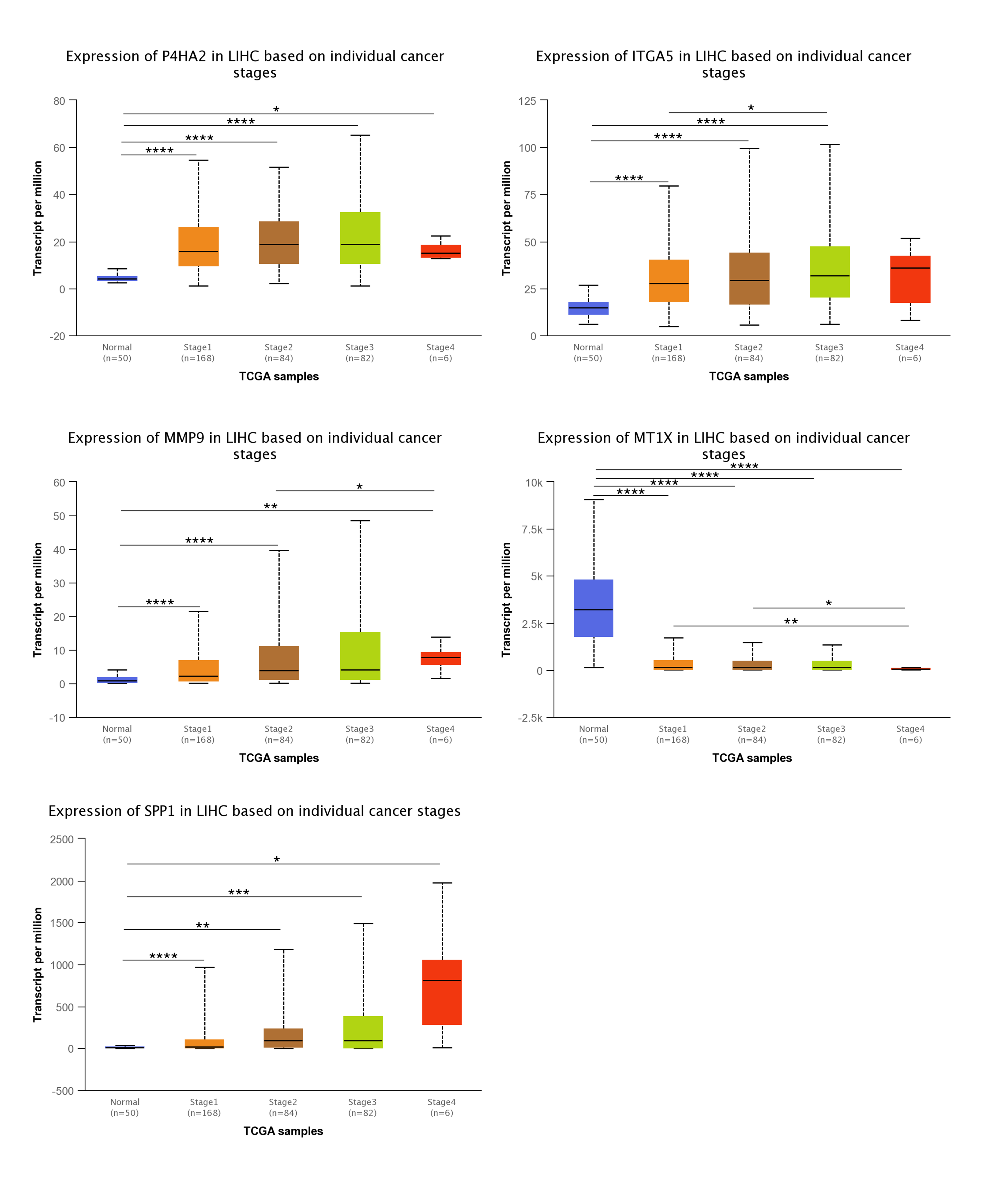

Supplement: Supplementary file 4 — Fig S4 [file JCMM-25-448-s004.tif]

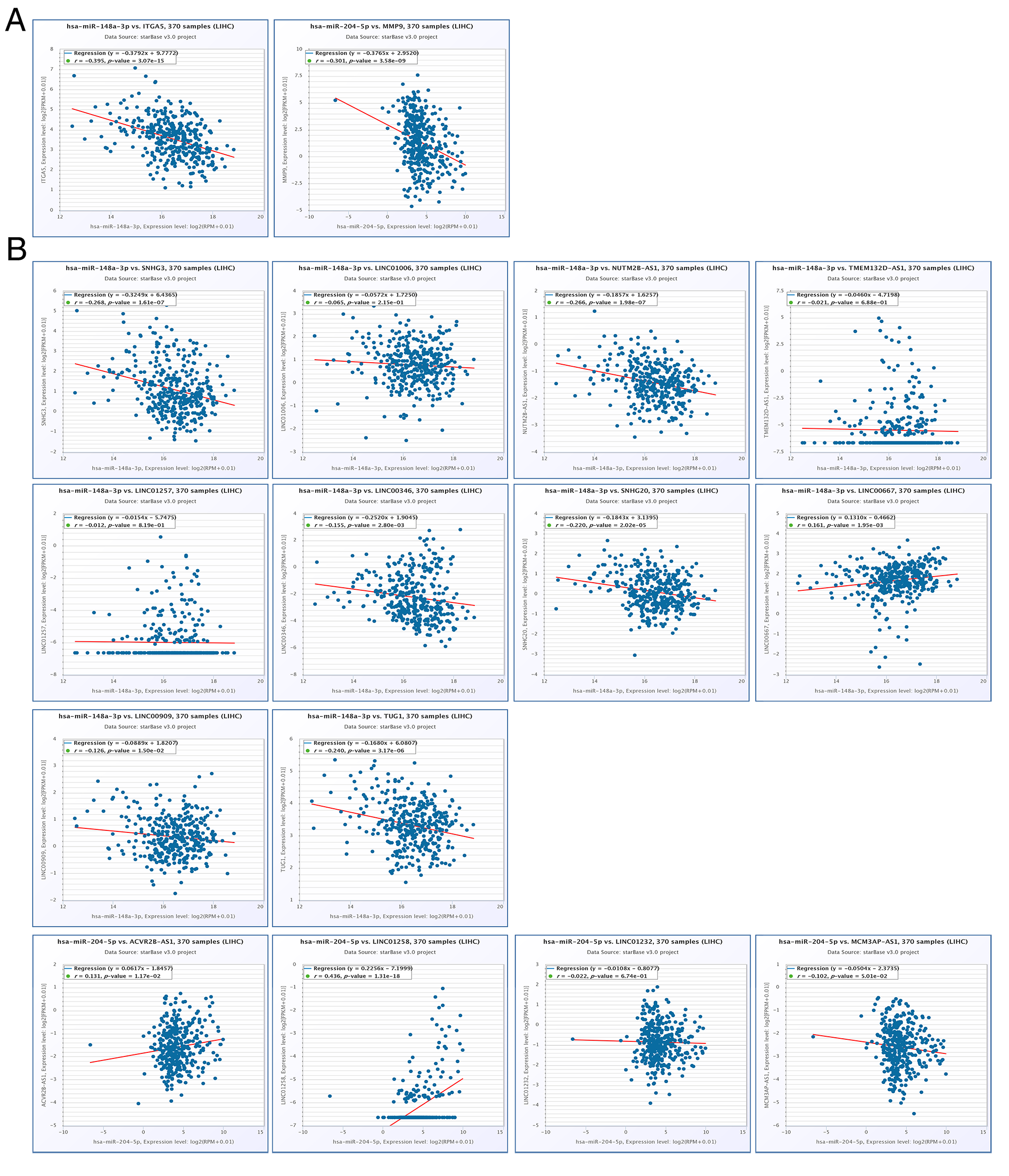

Supplement: Supplementary file 5 — Fig S5 [file JCMM-25-448-s005.tif]

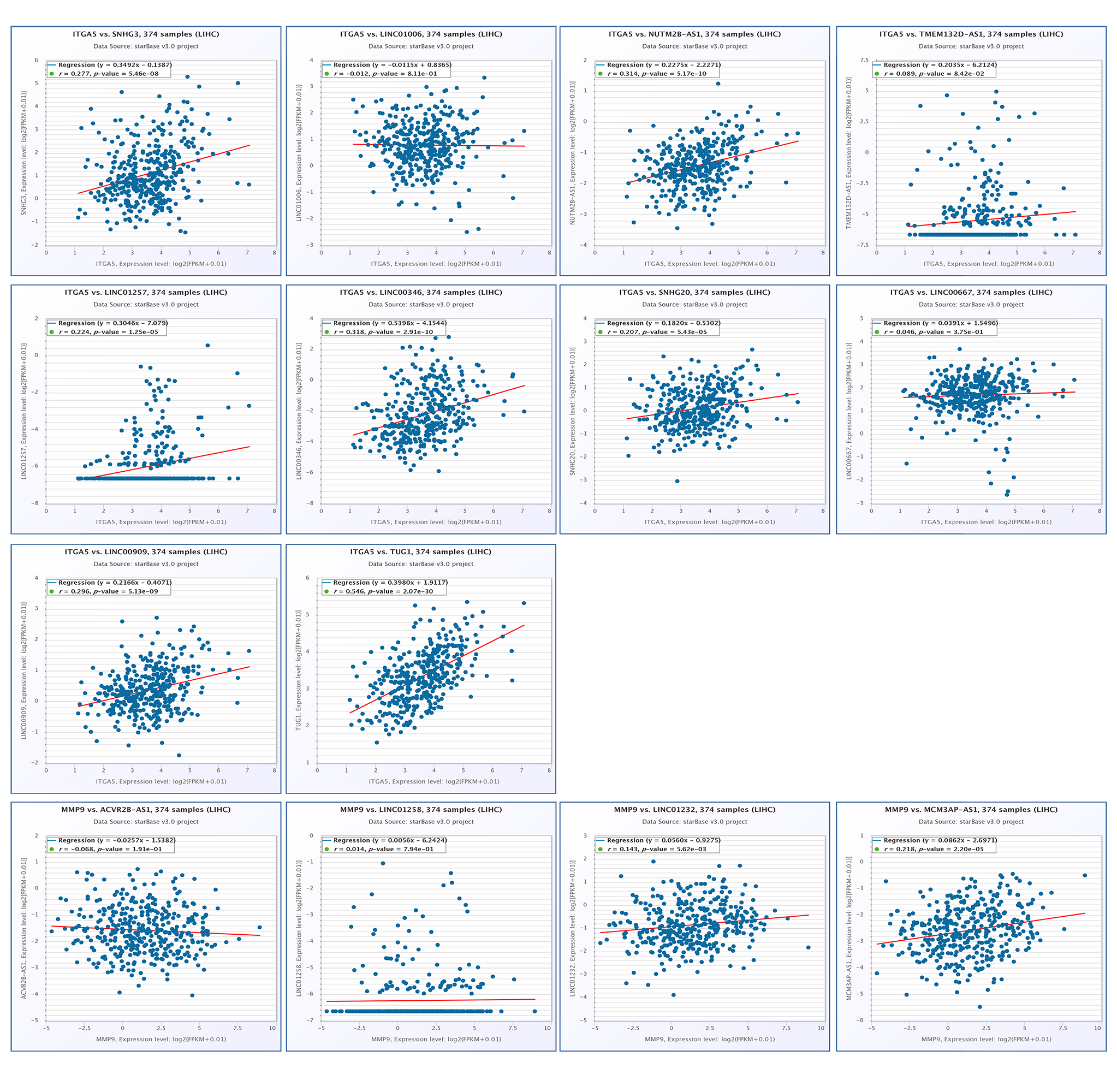

Supplement: Supplementary file 6 — Fig S6 [file JCMM-25-448-s006.tif]

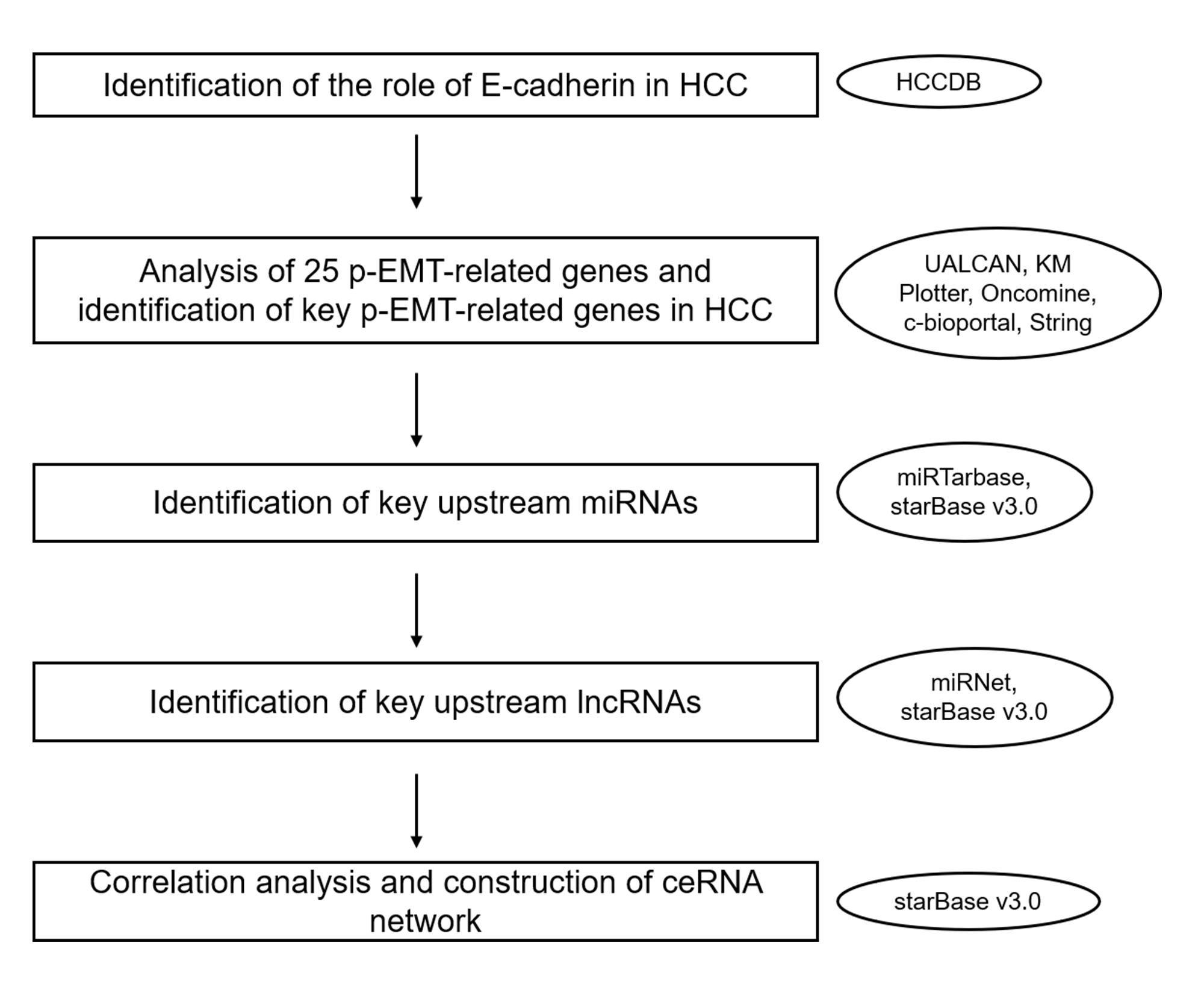

Supplement: Supplementary file 7 — Fig S7 [file JCMM-25-448-s007.tif]

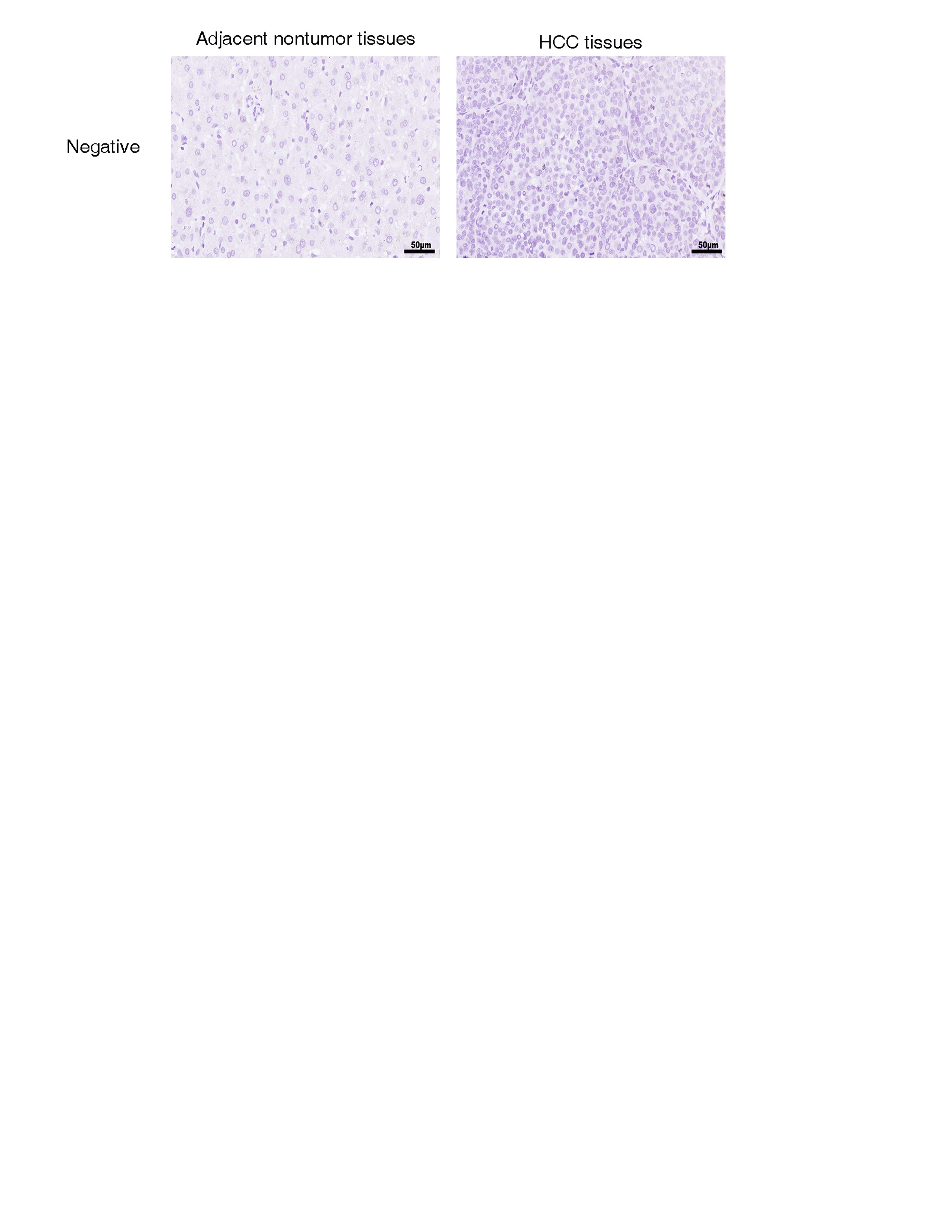

Supplement: Supplementary file 8 — Fig S8 [file JCMM-25-448-s008.tif]

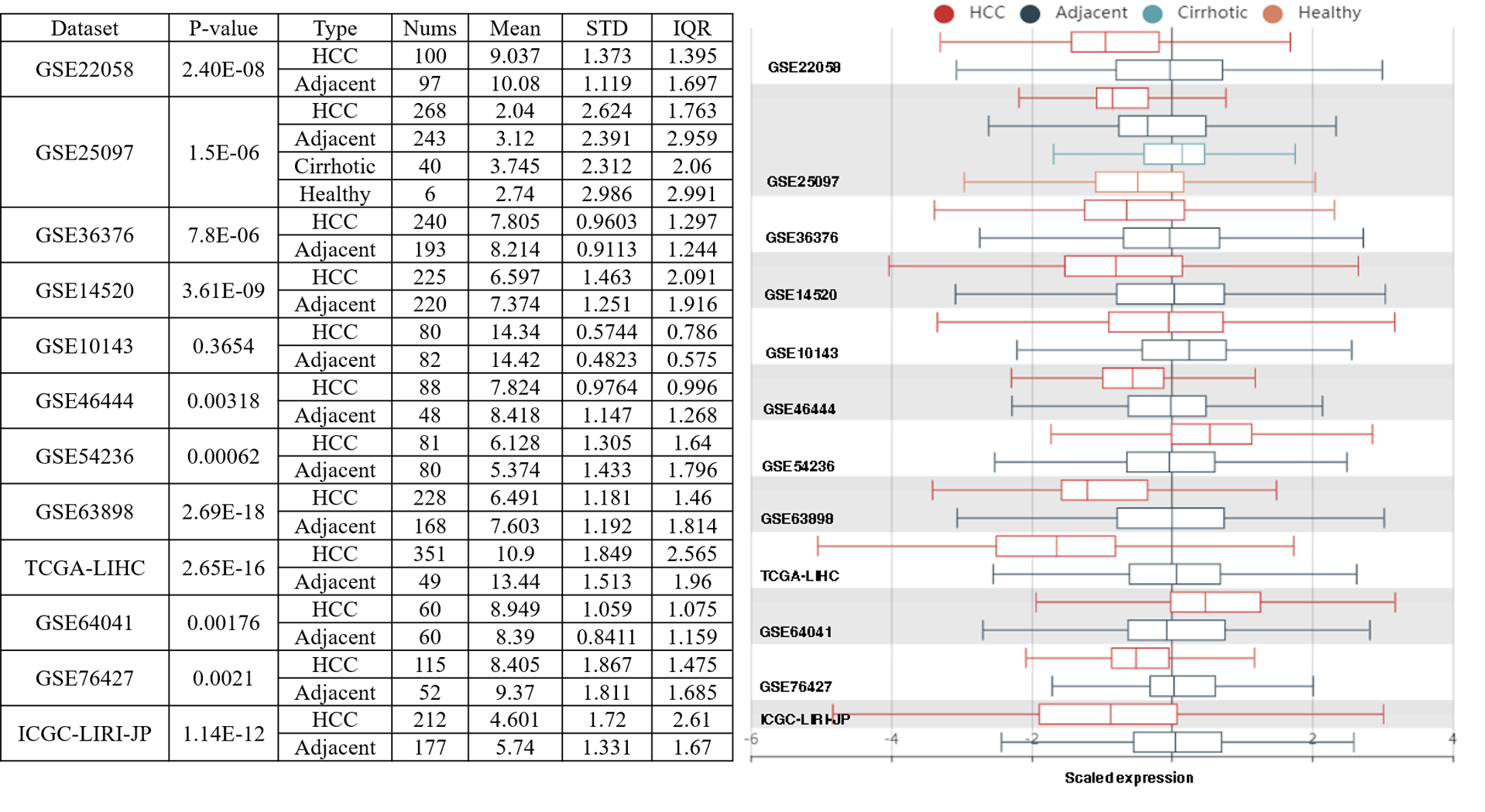

Supplement: Supplementary file 9 — Fig S9 [file JCMM-25-448-s009.tif]

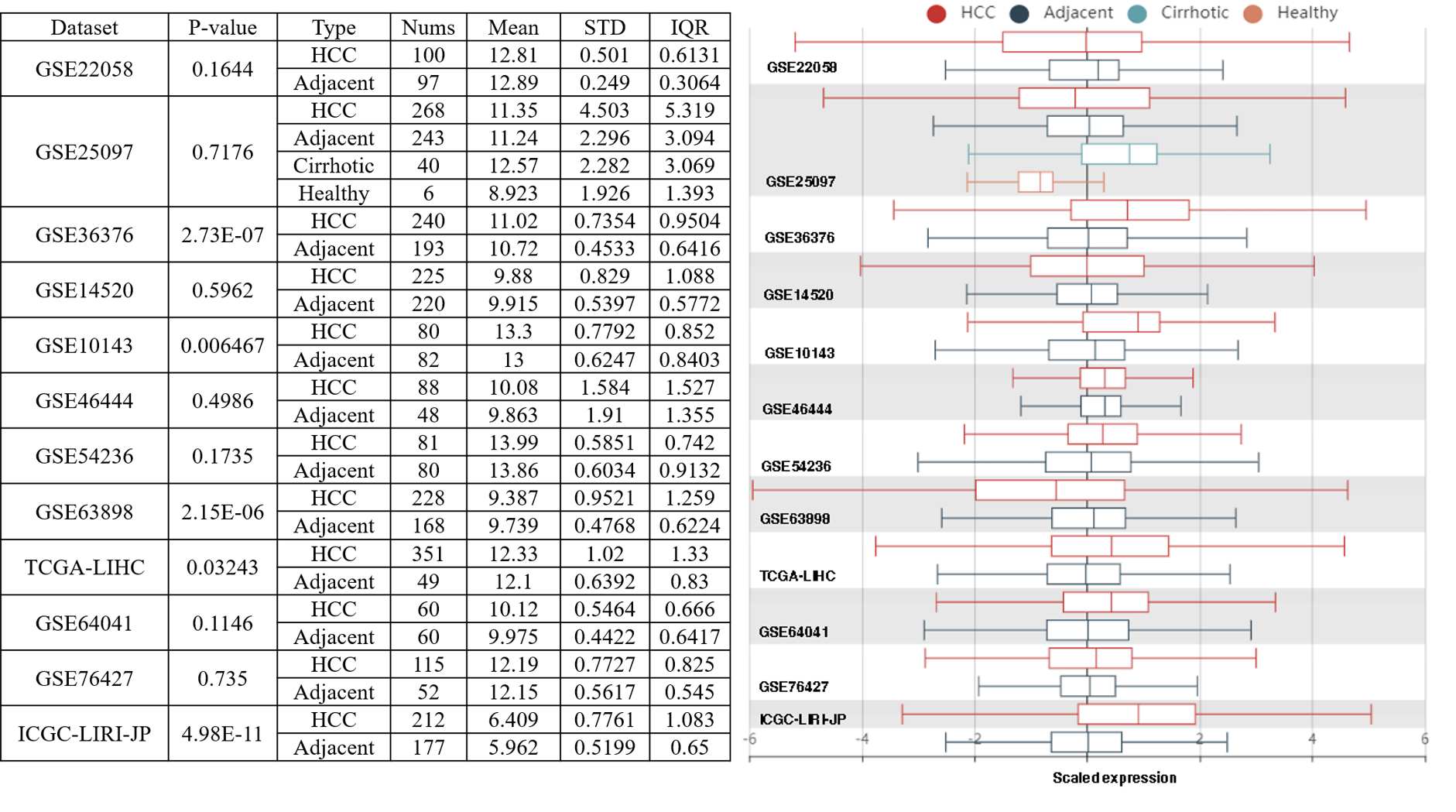

Supplement: Supplementary file 10 — Fig S10 [file JCMM-25-448-s010.tif]

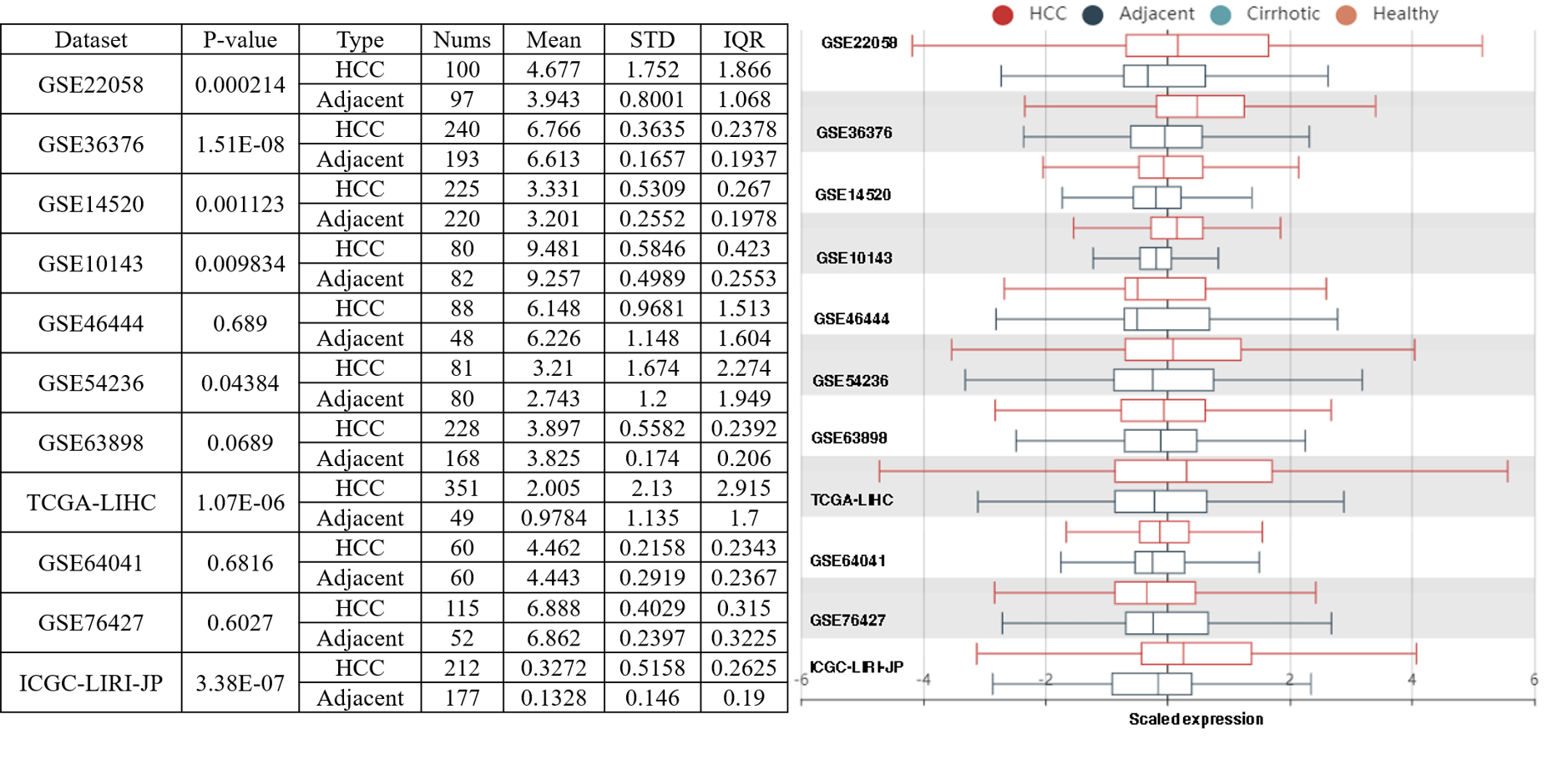

Supplement: Supplementary file 11 — Fig S11 [file JCMM-25-448-s011.tif]

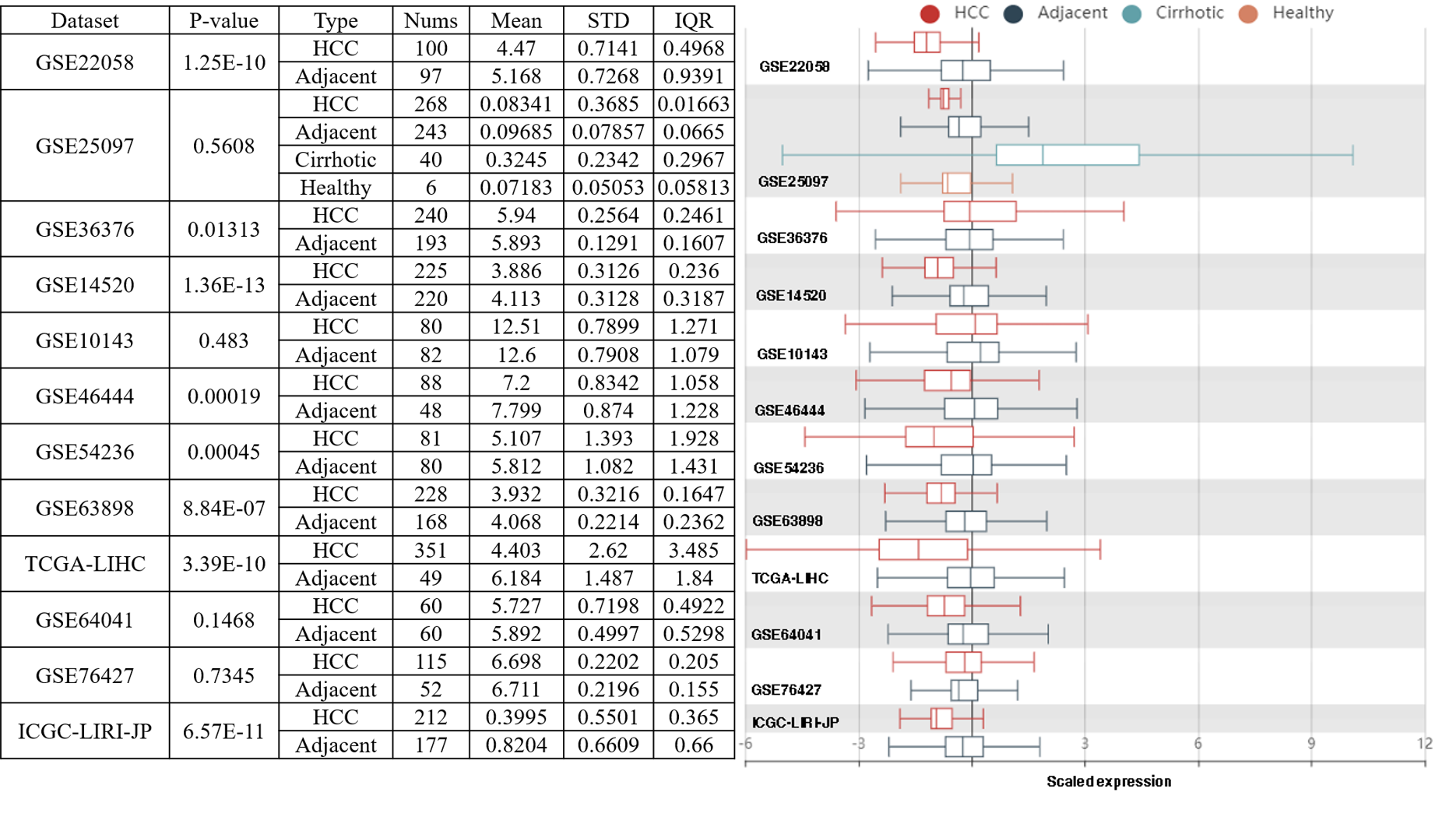

Supplement: Supplementary file 12 — Fig S12 [file JCMM-25-448-s012.tif]

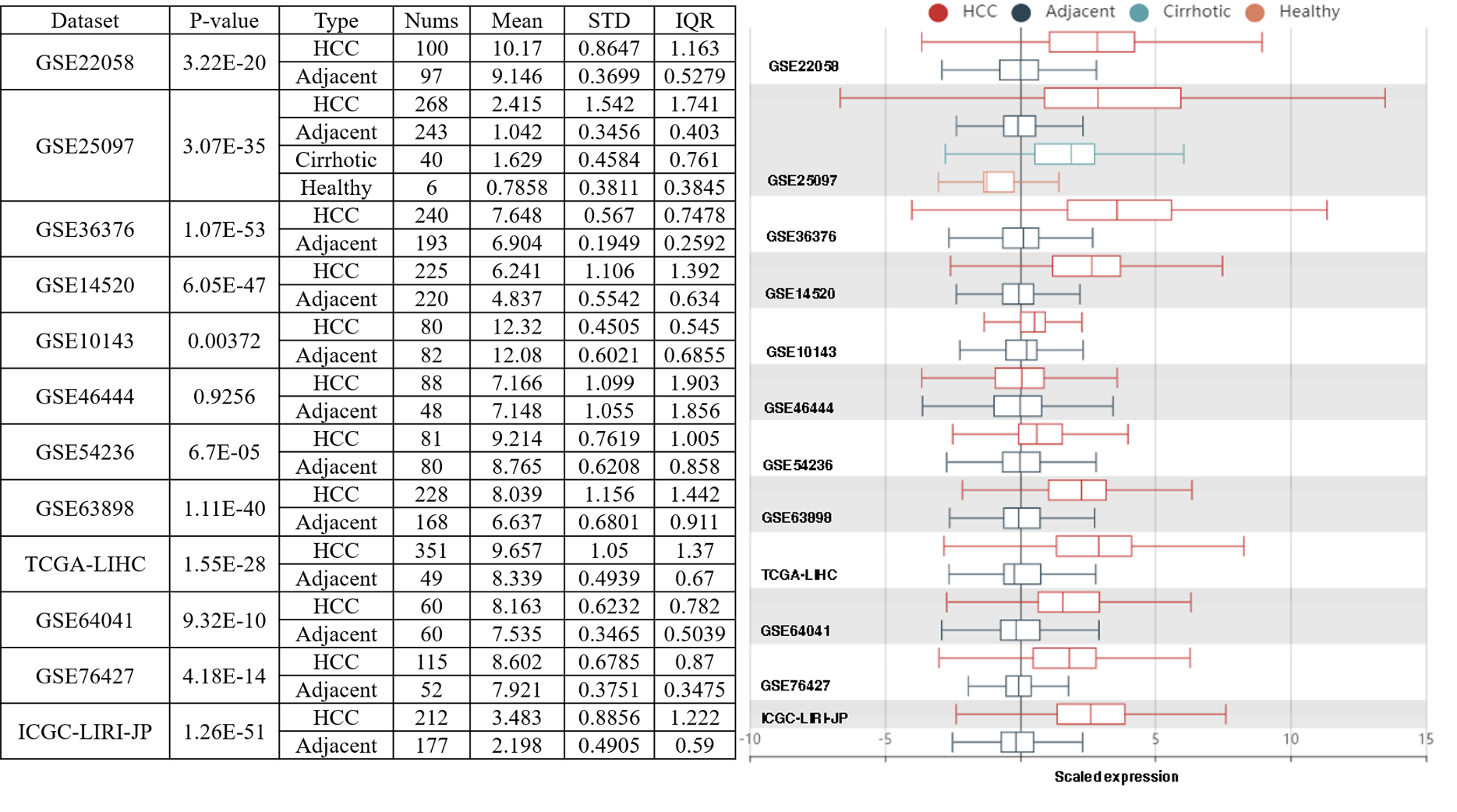

Supplement: Supplementary file 13 — Fig S13 [file JCMM-25-448-s013.tif]

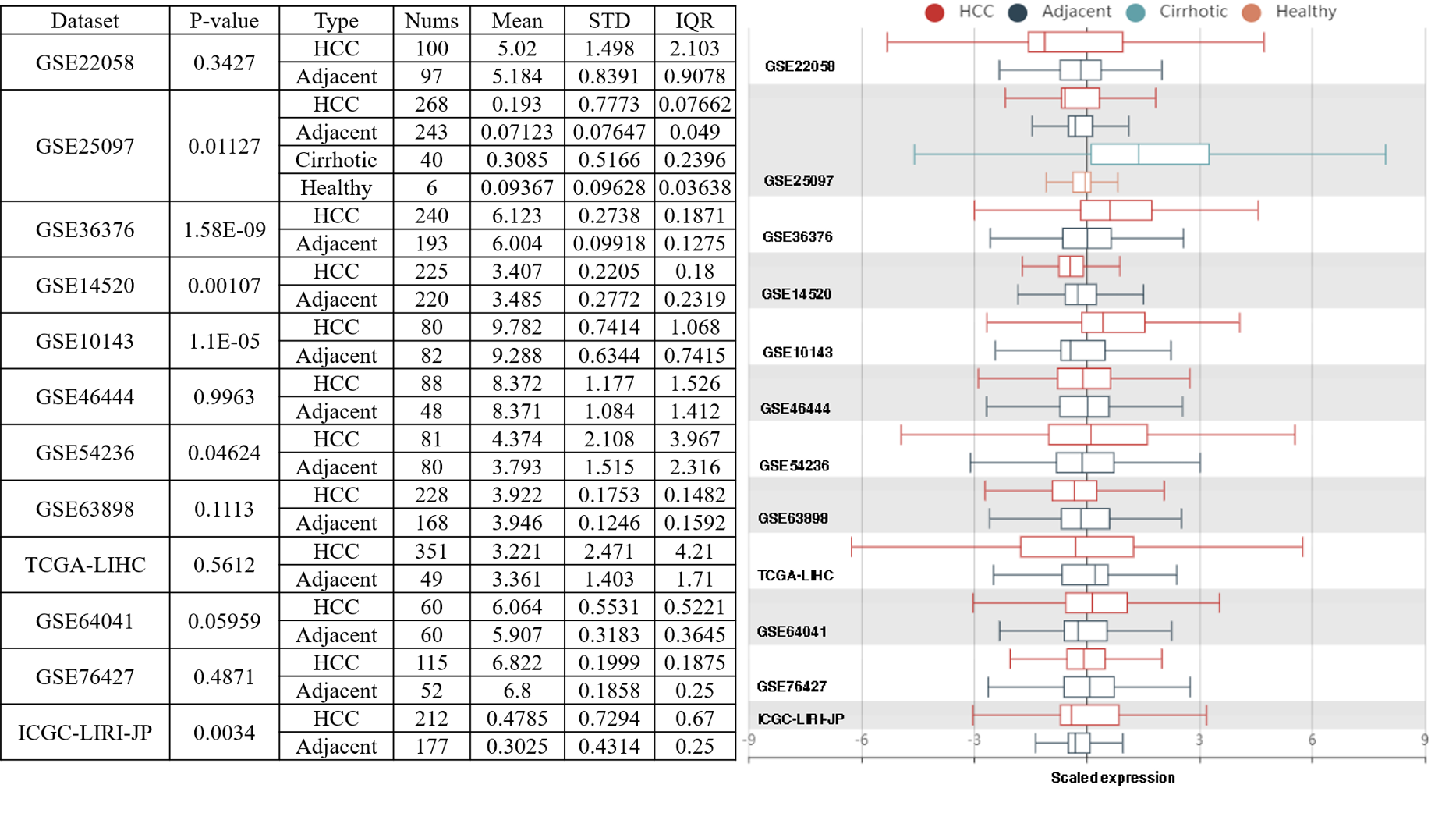

Supplement: Supplementary file 14 — Fig S14 [file JCMM-25-448-s014.tif]

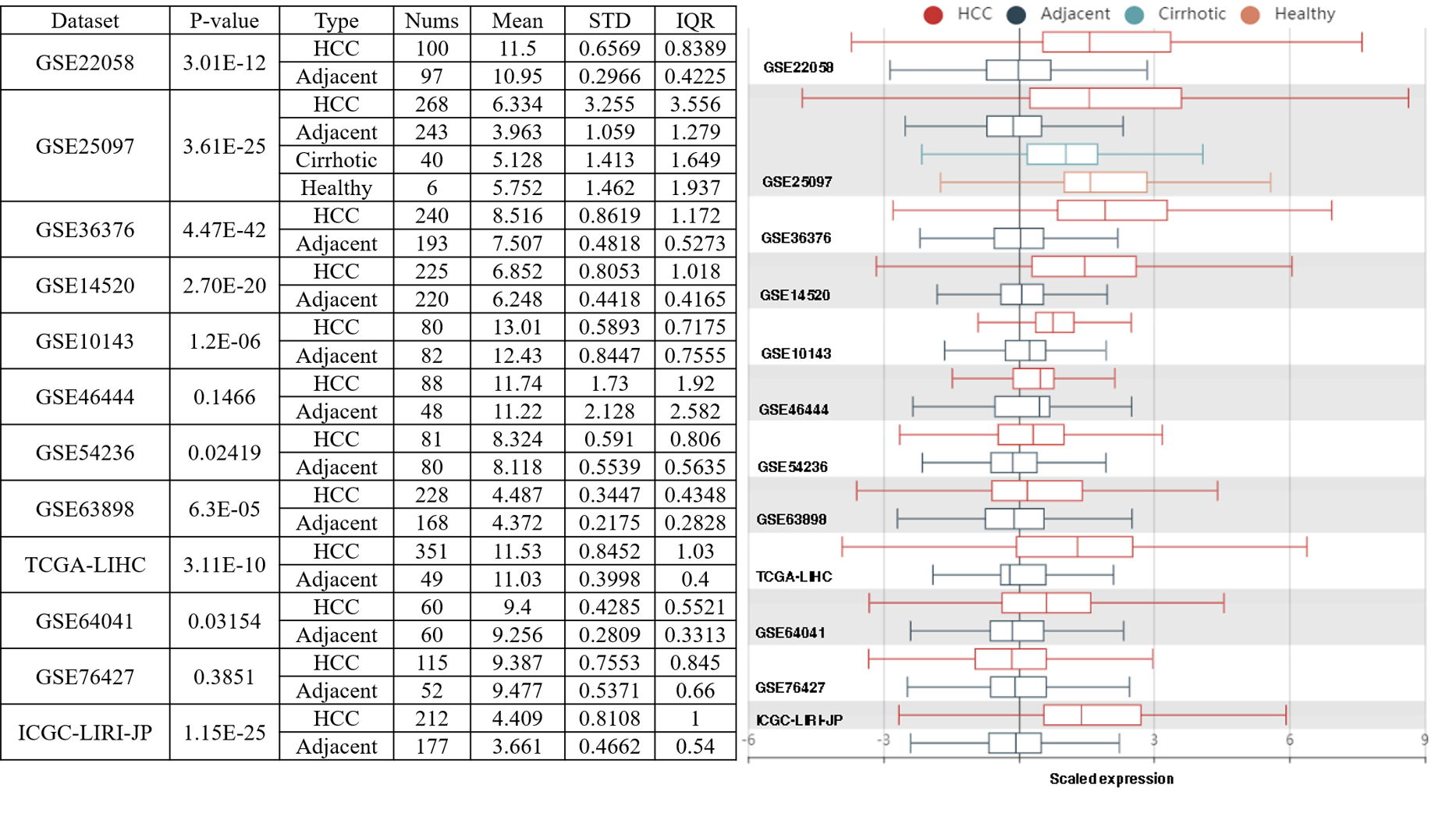

Supplement: Supplementary file 15 — Fig S15 [file JCMM-25-448-s015.tif]

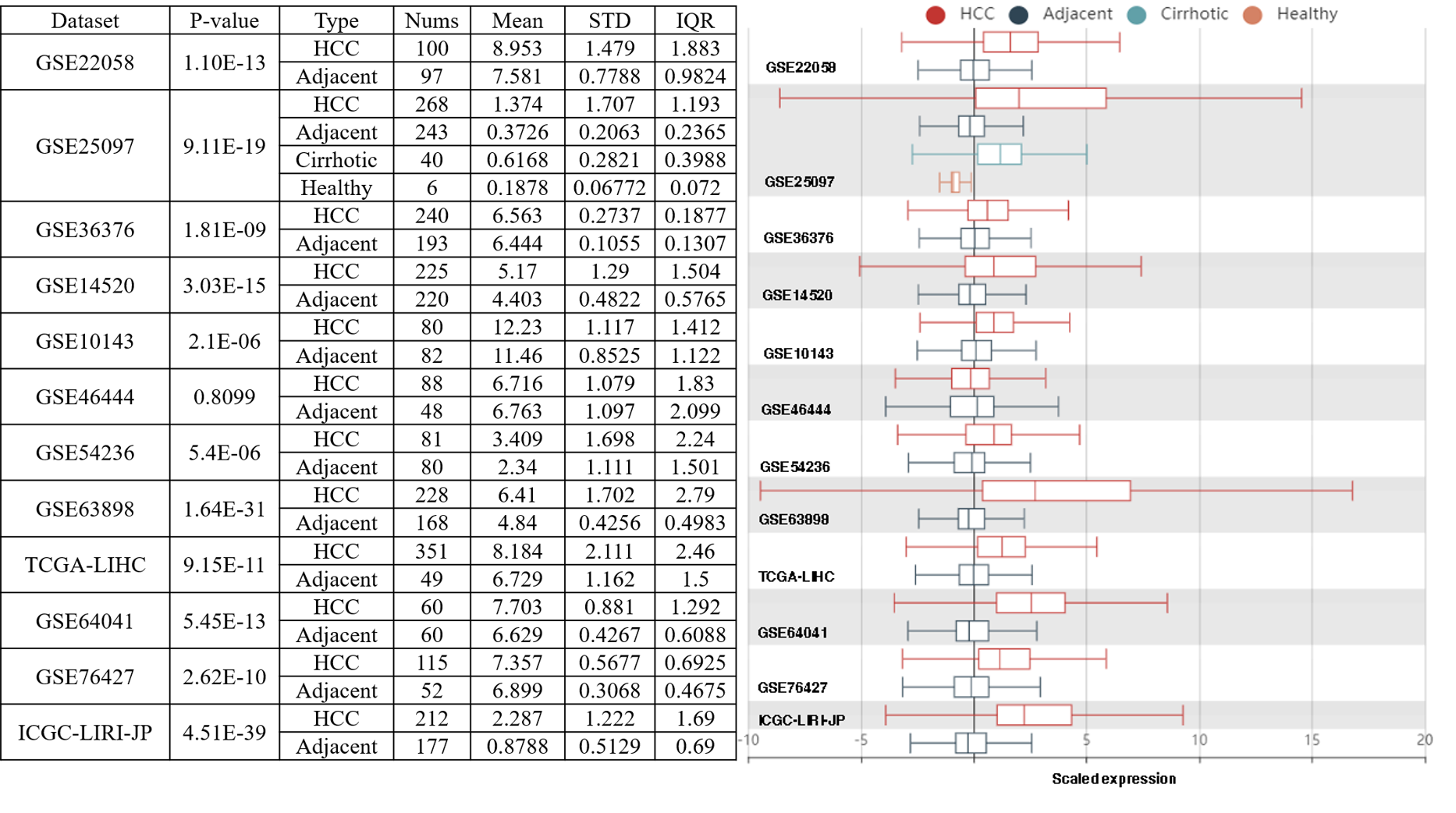

Supplement: Supplementary file 16 — Fig S16 [file JCMM-25-448-s016.tif]

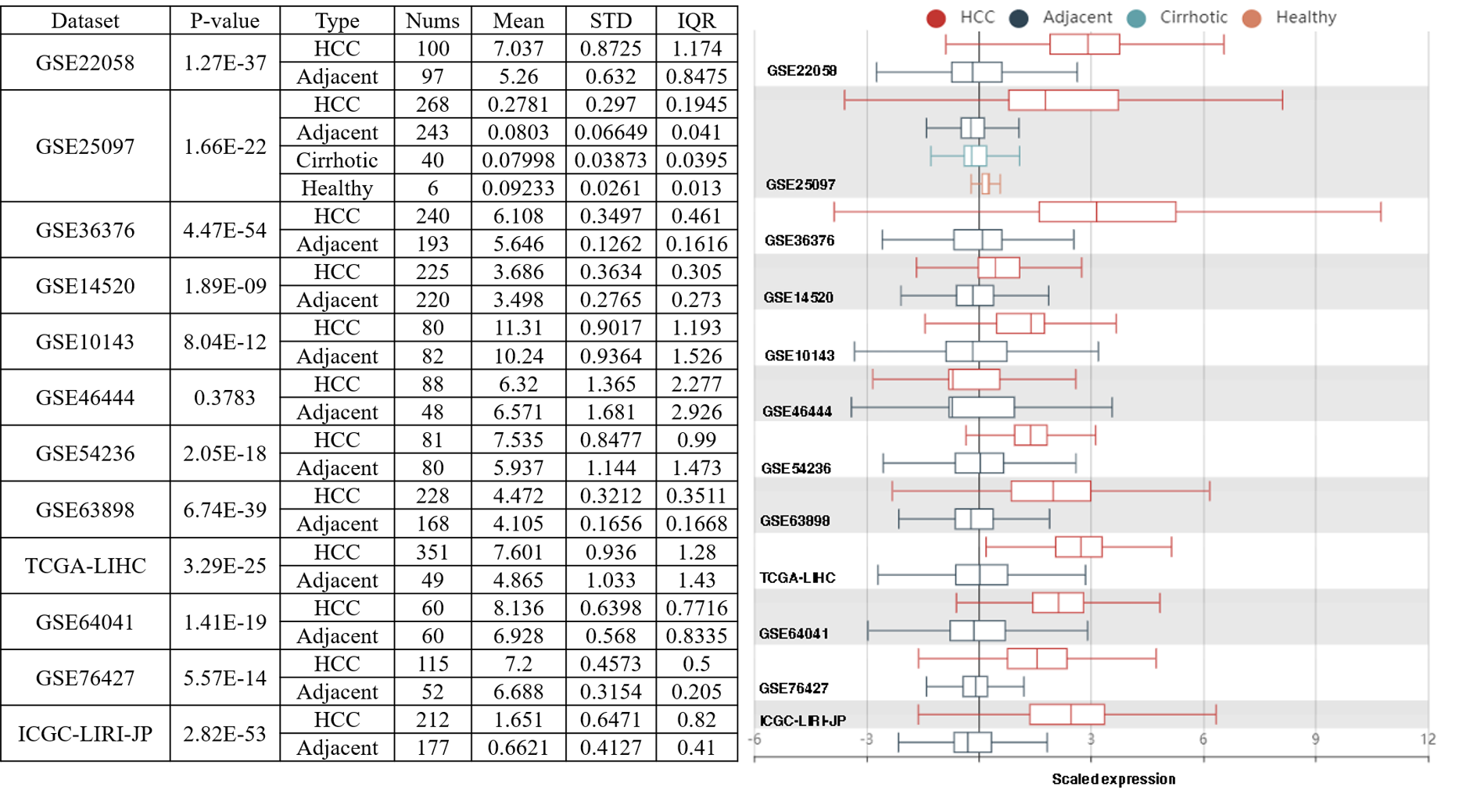

Supplement: Supplementary file 17 — fig S17 [file JCMM-25-448-s017.tif]

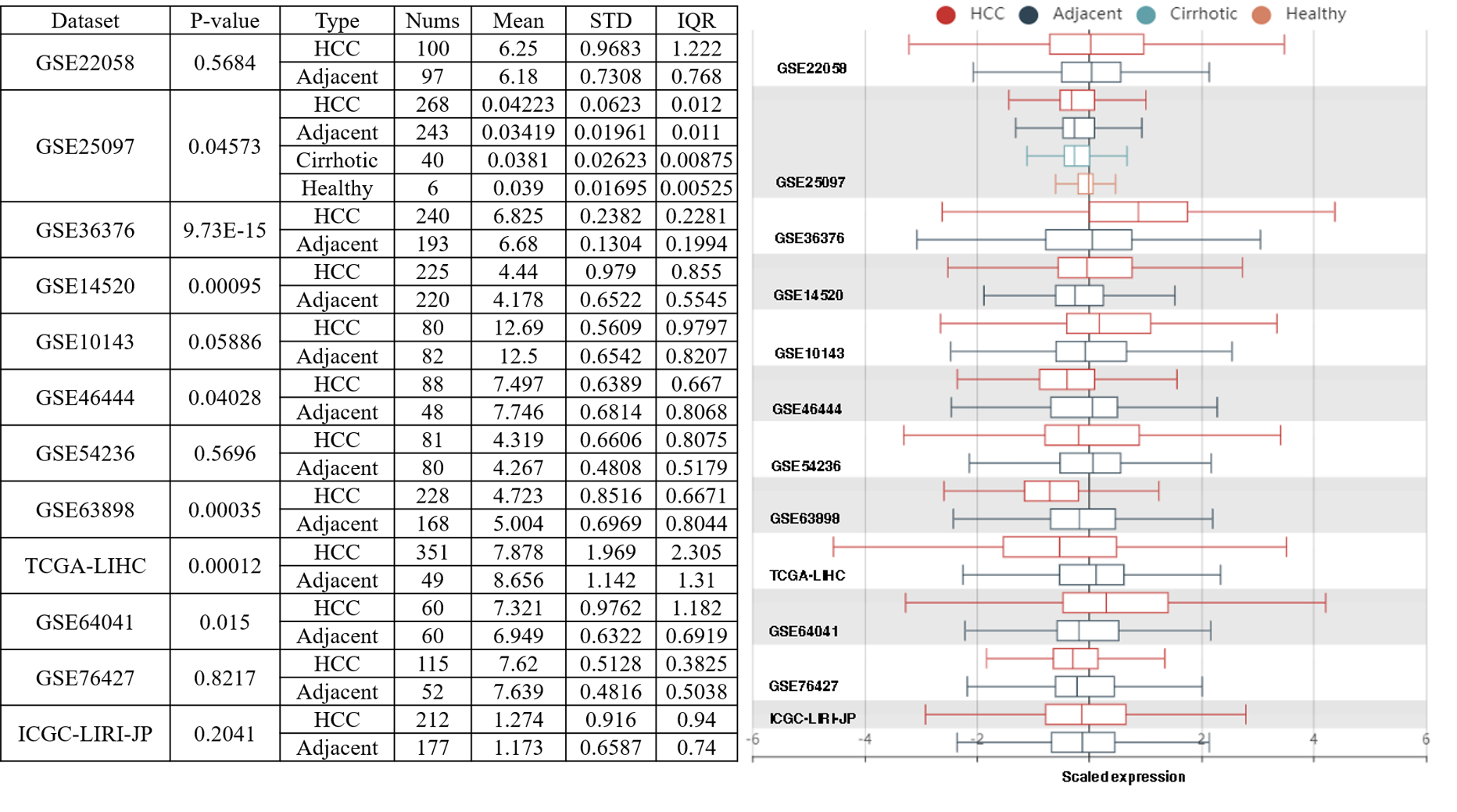

Supplement: Supplementary file 18 — Fig S18 [file JCMM-25-448-s018.tif]

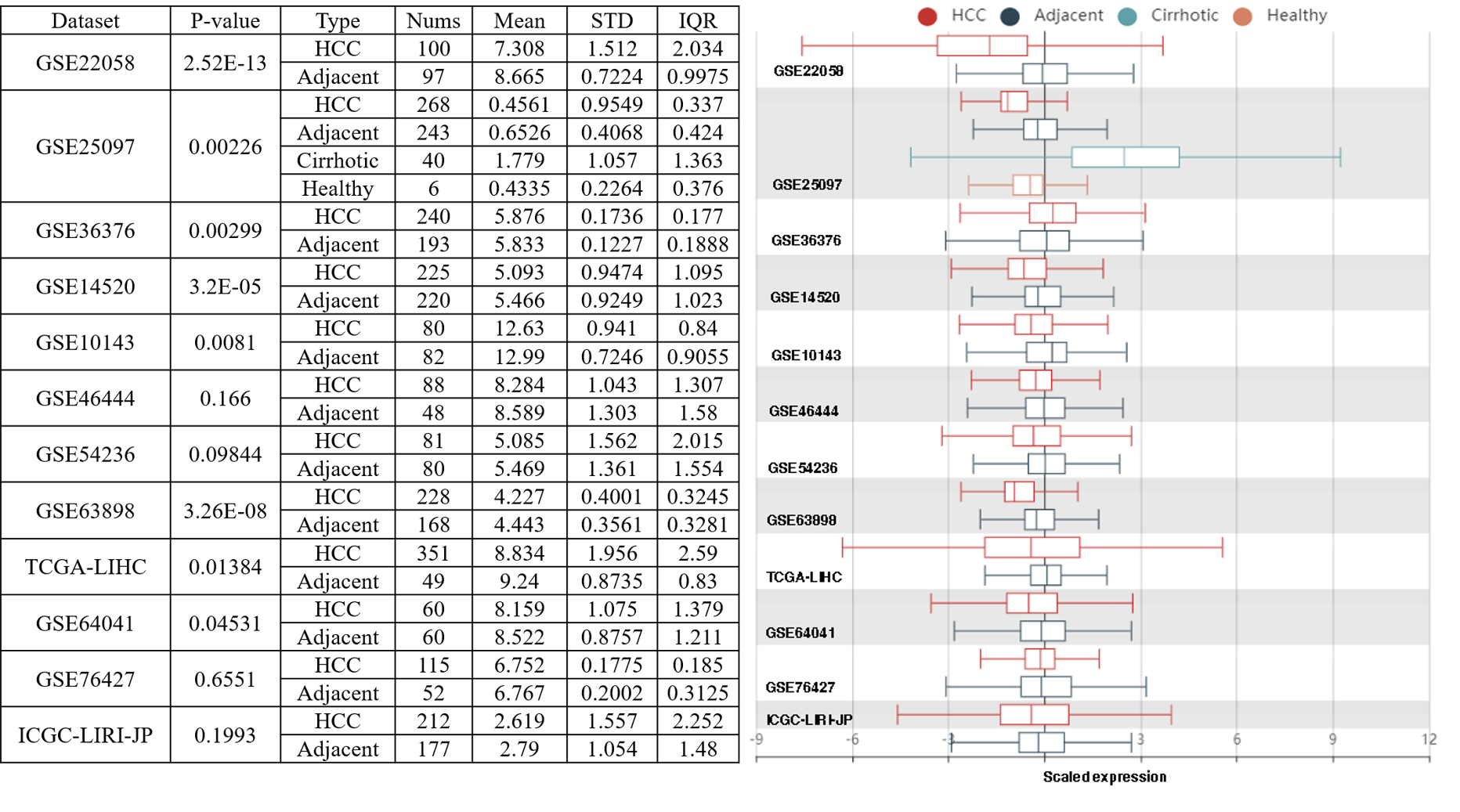

Supplement: Supplementary file 19 — Fig S19 [file JCMM-25-448-s019.tif]

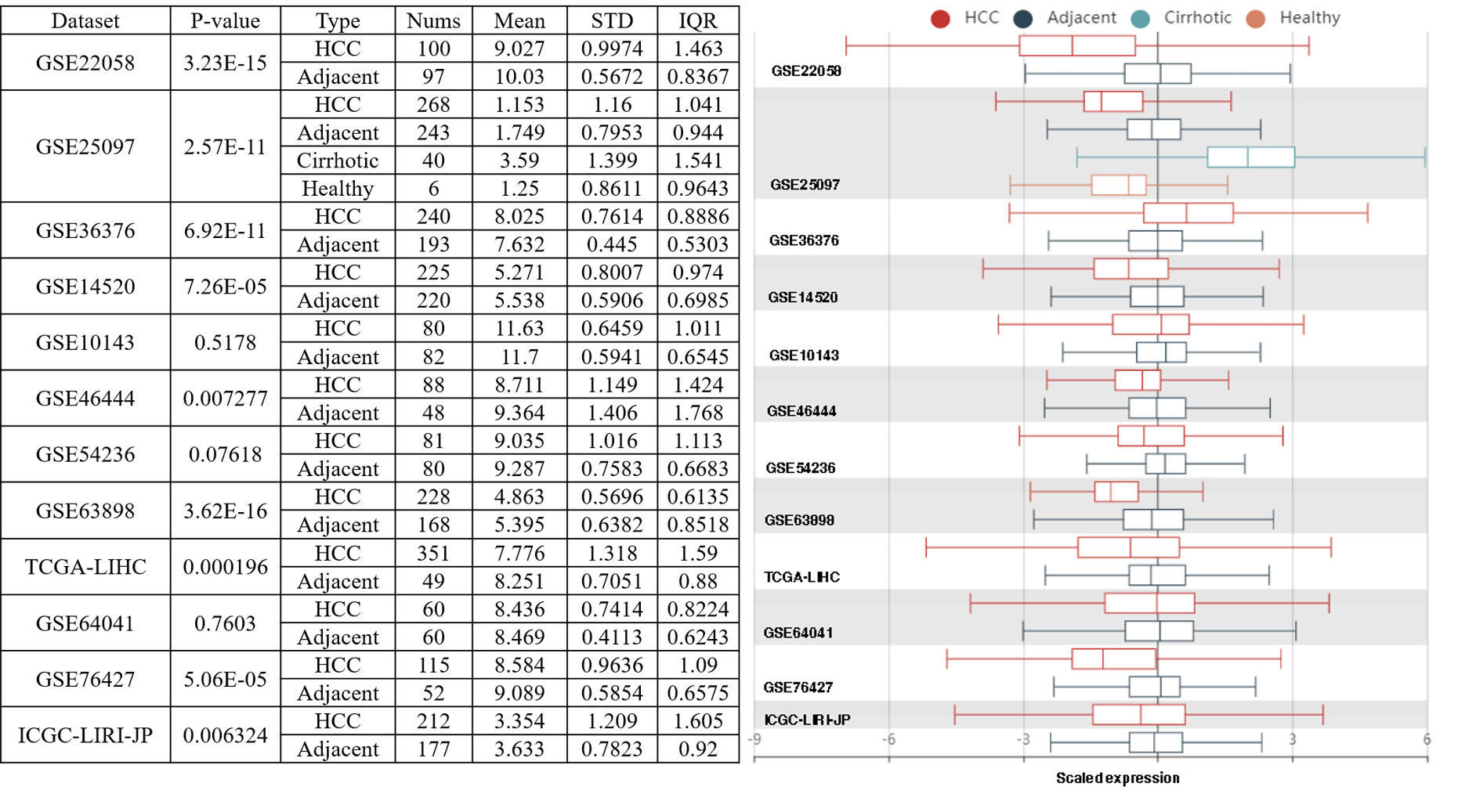

Supplement: Supplementary file 20 — Fig S20 [file JCMM-25-448-s020.tif]

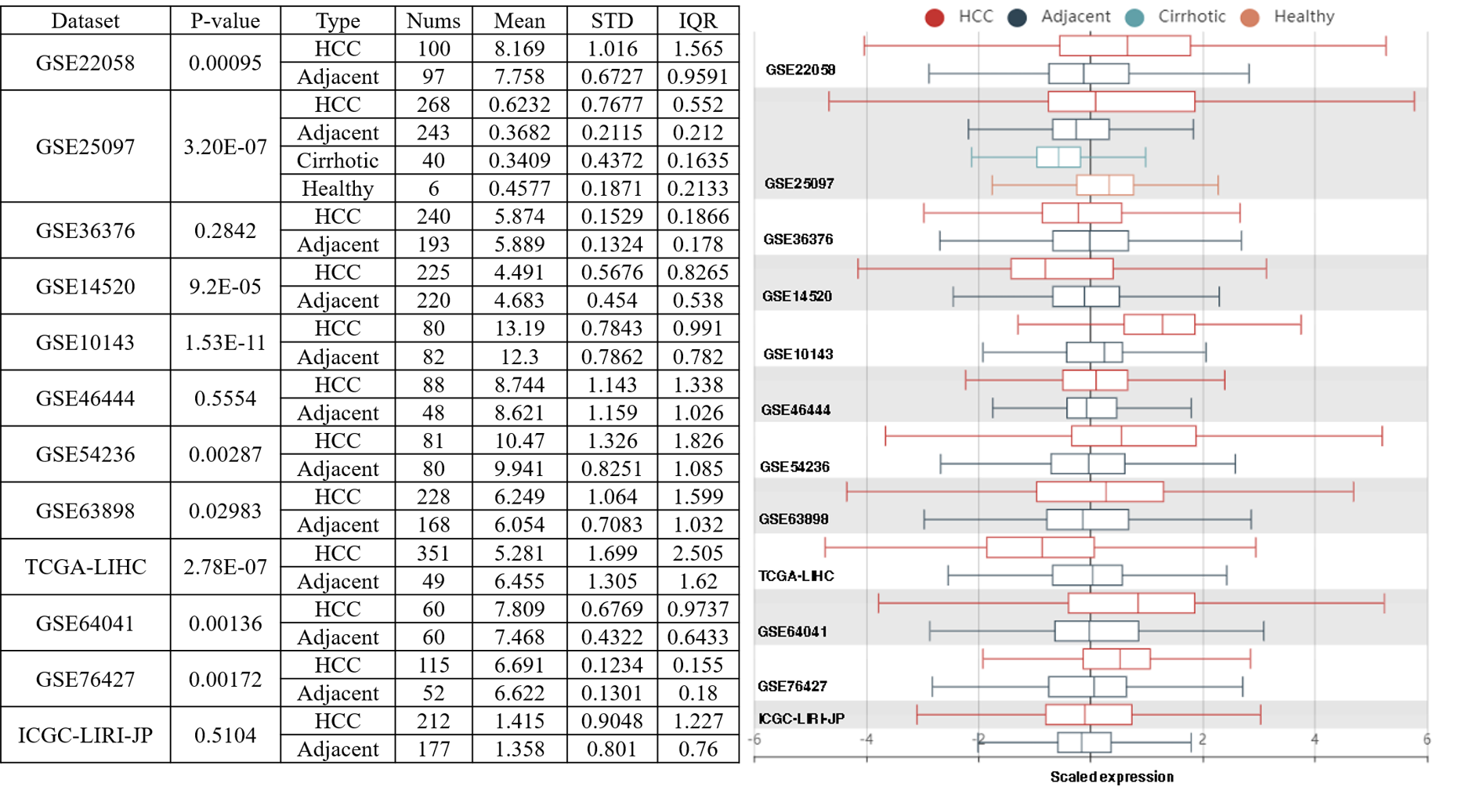

Supplement: Supplementary file 21 — Fig S21 [file JCMM-25-448-s021.tif]

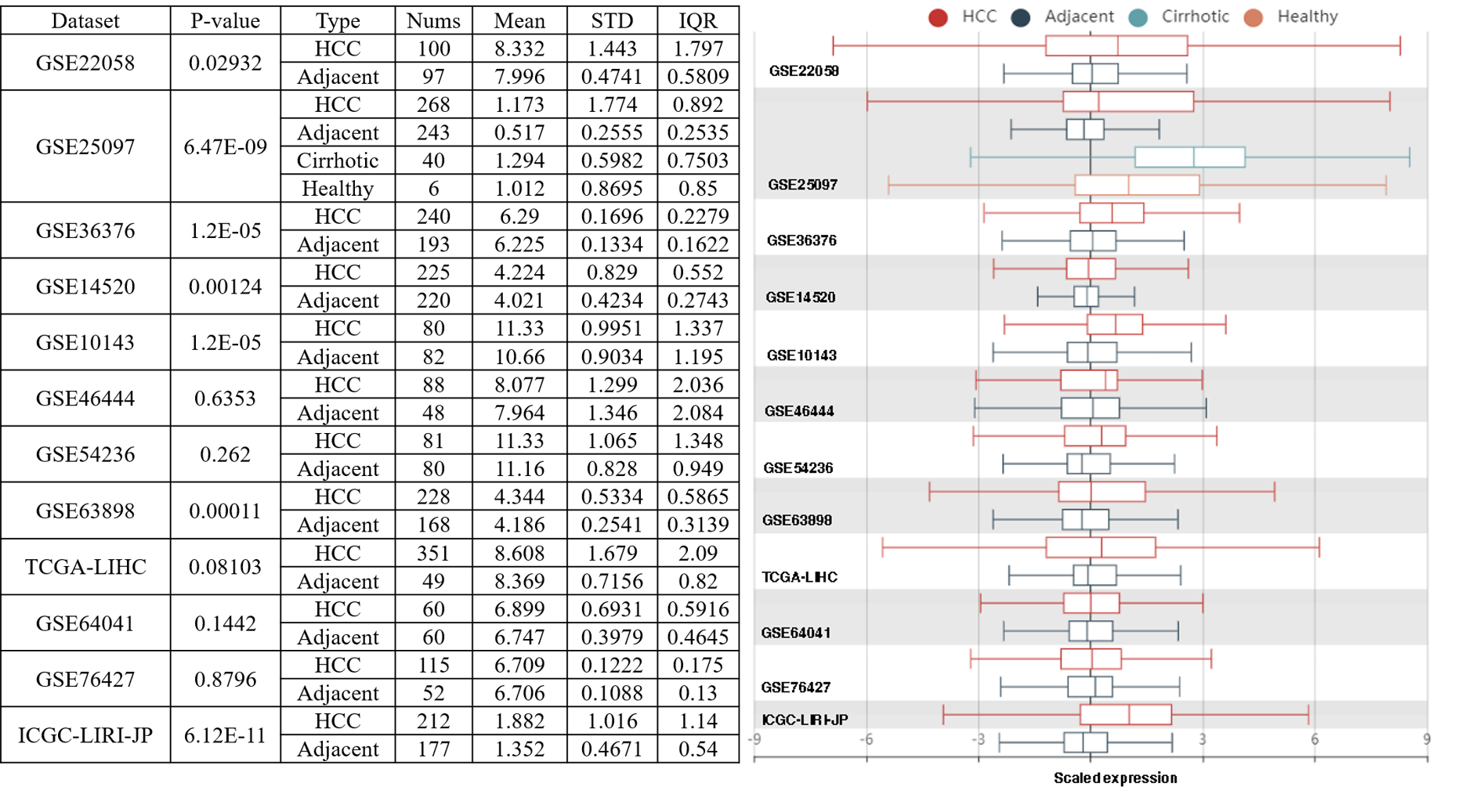

Supplement: Supplementary file 22 — Fig S22 [file JCMM-25-448-s022.tif]

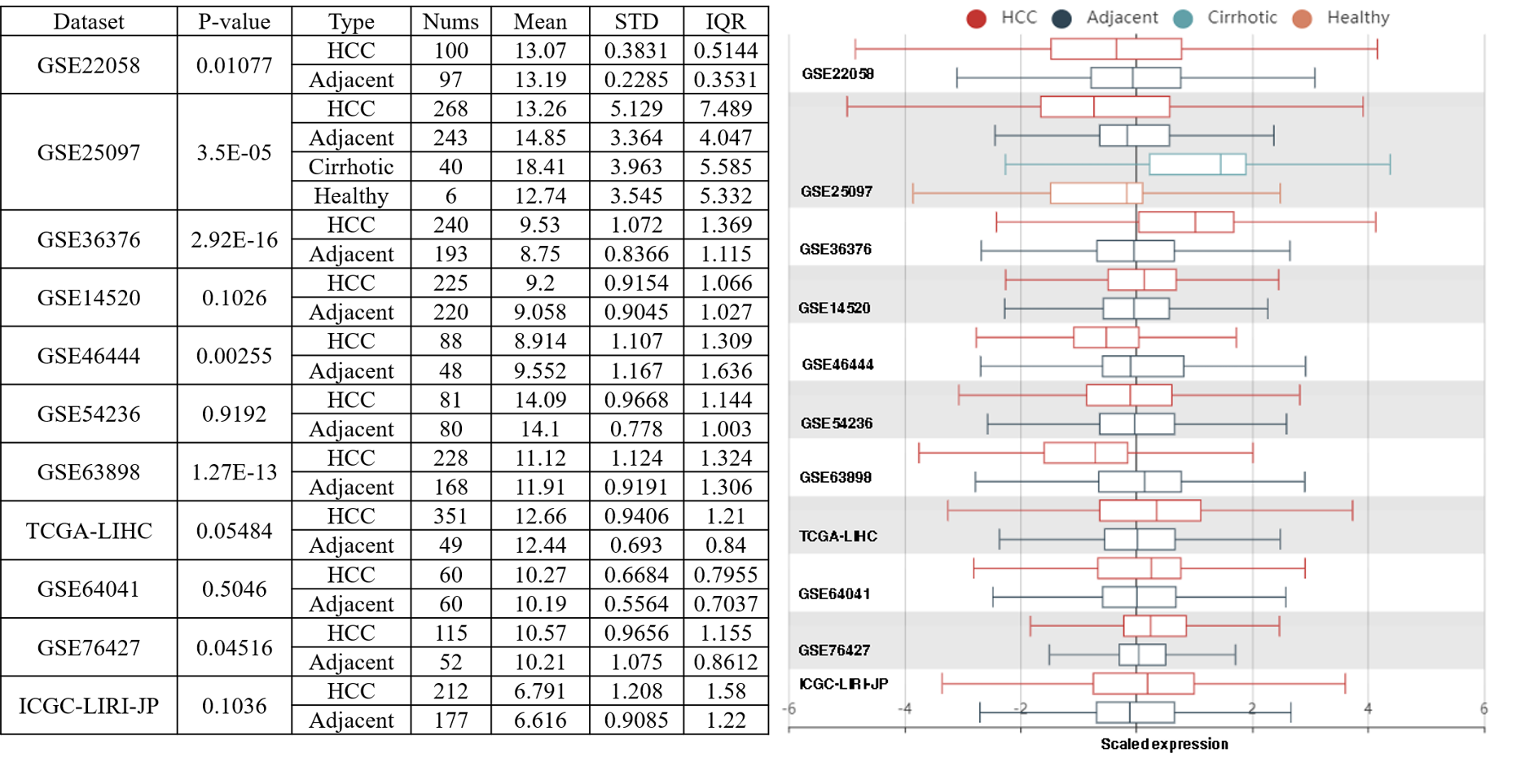

Supplement: Supplementary file 23 — Fig S23 [file JCMM-25-448-s023.tif]

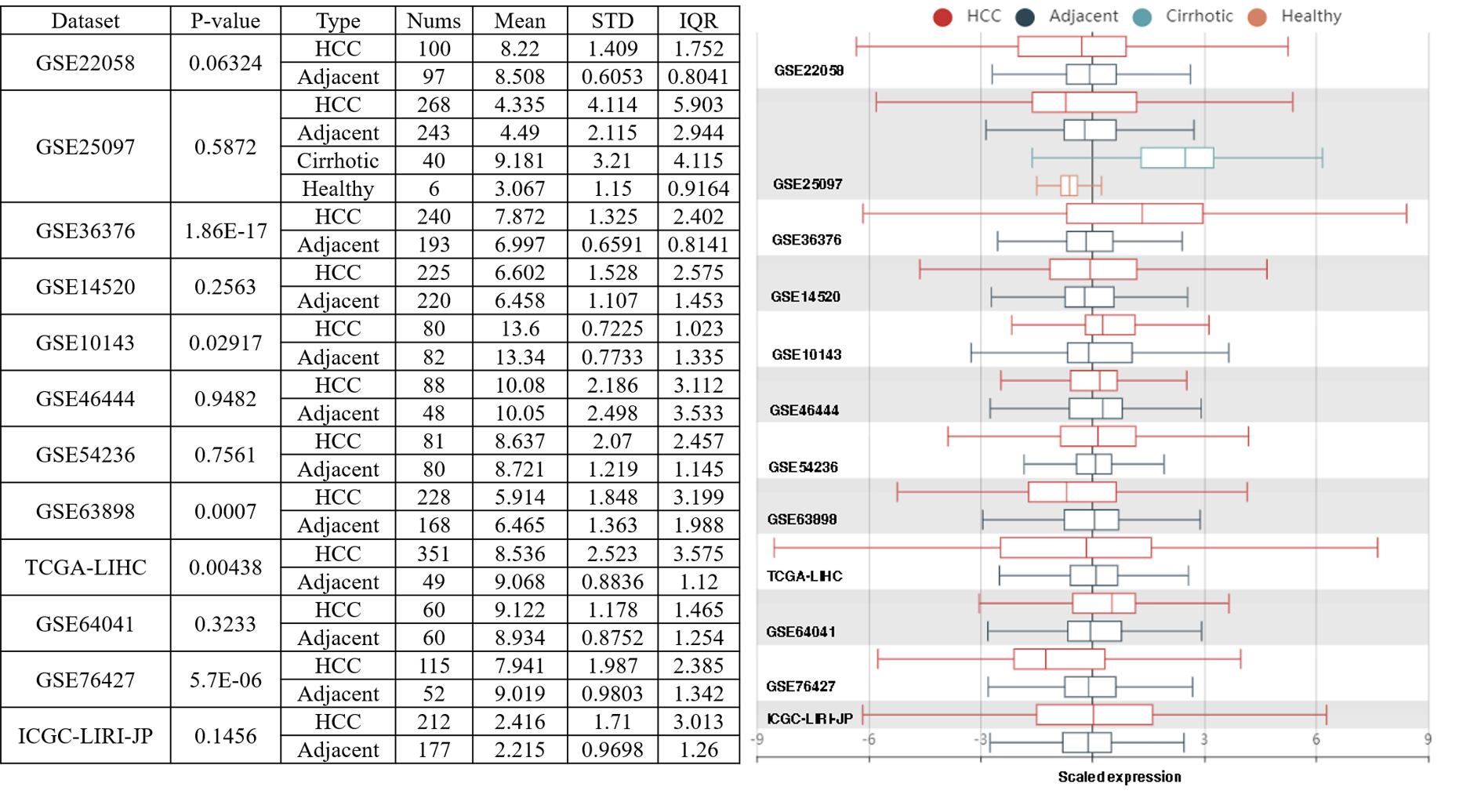

Supplement: Supplementary file 24 — Fig S24 [file JCMM-25-448-s024.tif]

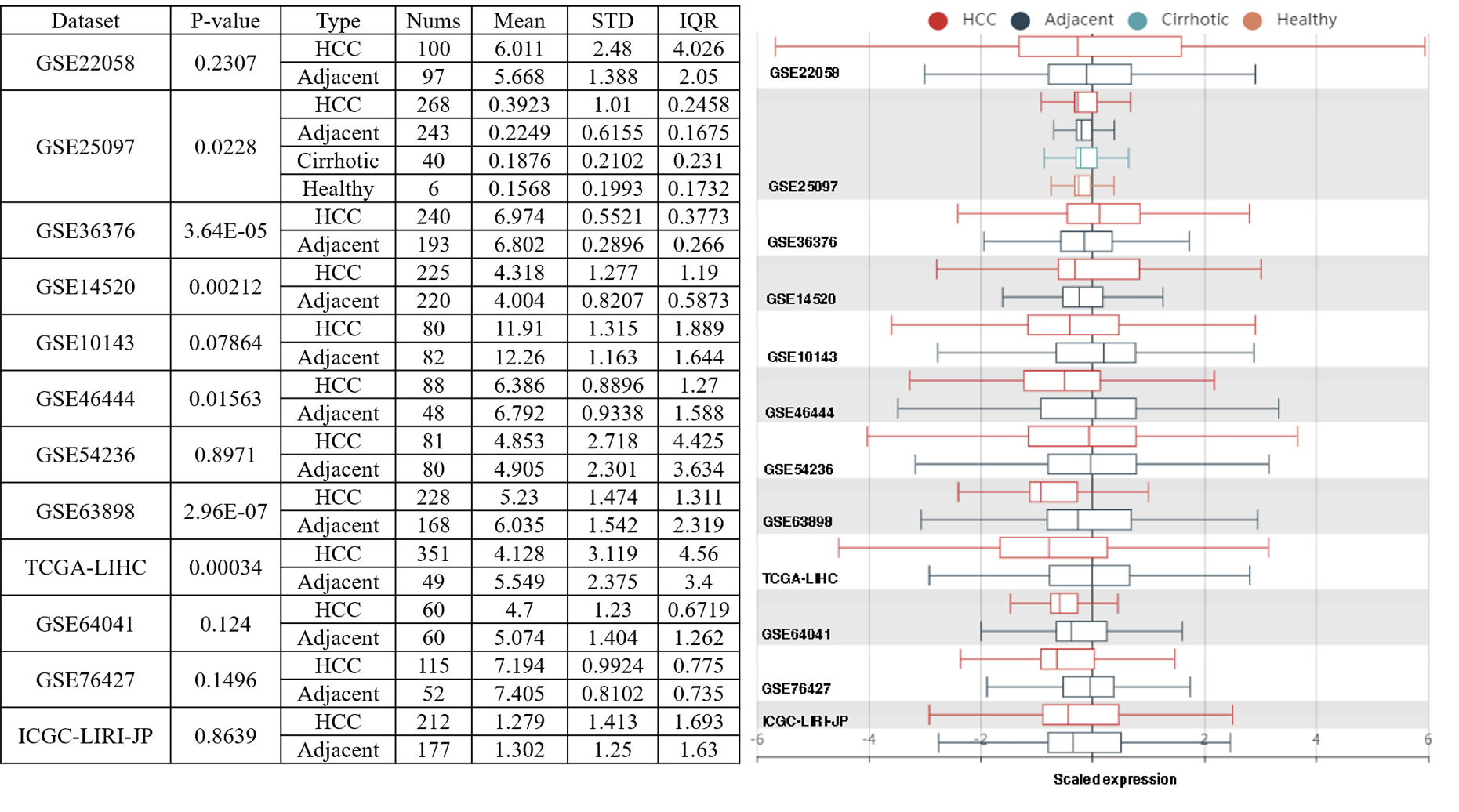

Supplement: Supplementary file 25 — Fig S25 [file JCMM-25-448-s025.tif]

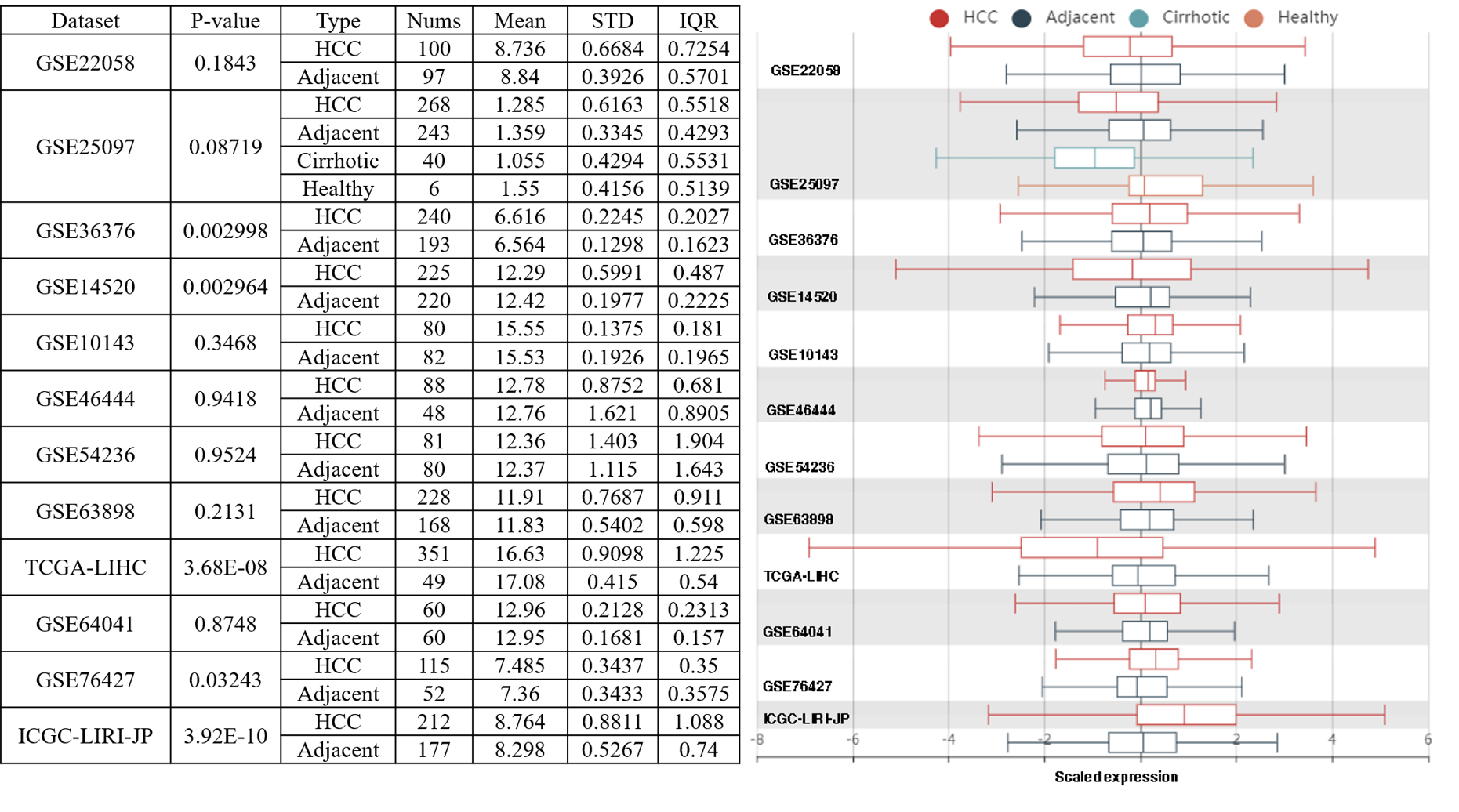

Supplement: Supplementary file 26 — Fig S26 [file JCMM-25-448-s026.tif]

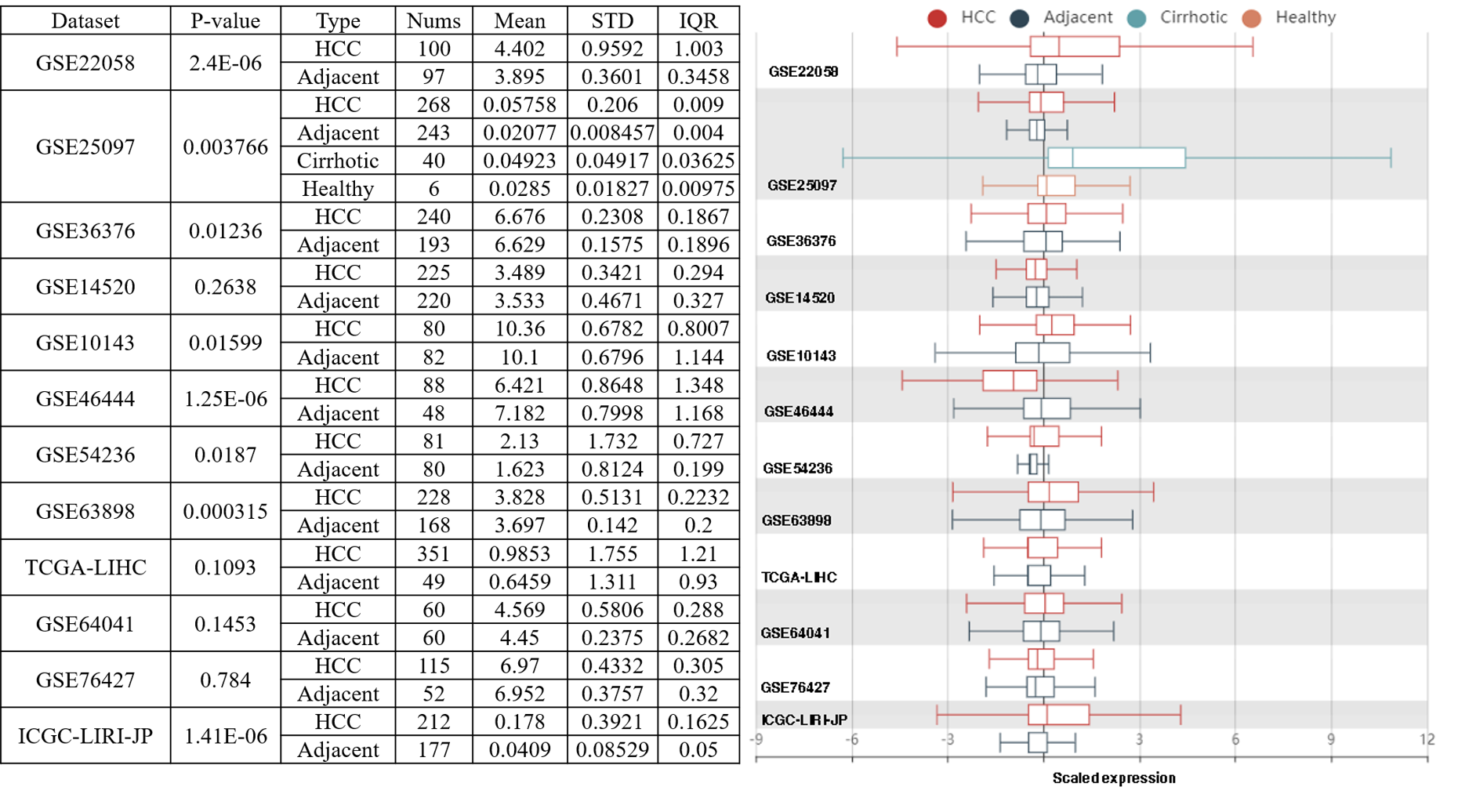

Supplement: Supplementary file 27 — Fig S27 [file JCMM-25-448-s027.tif]

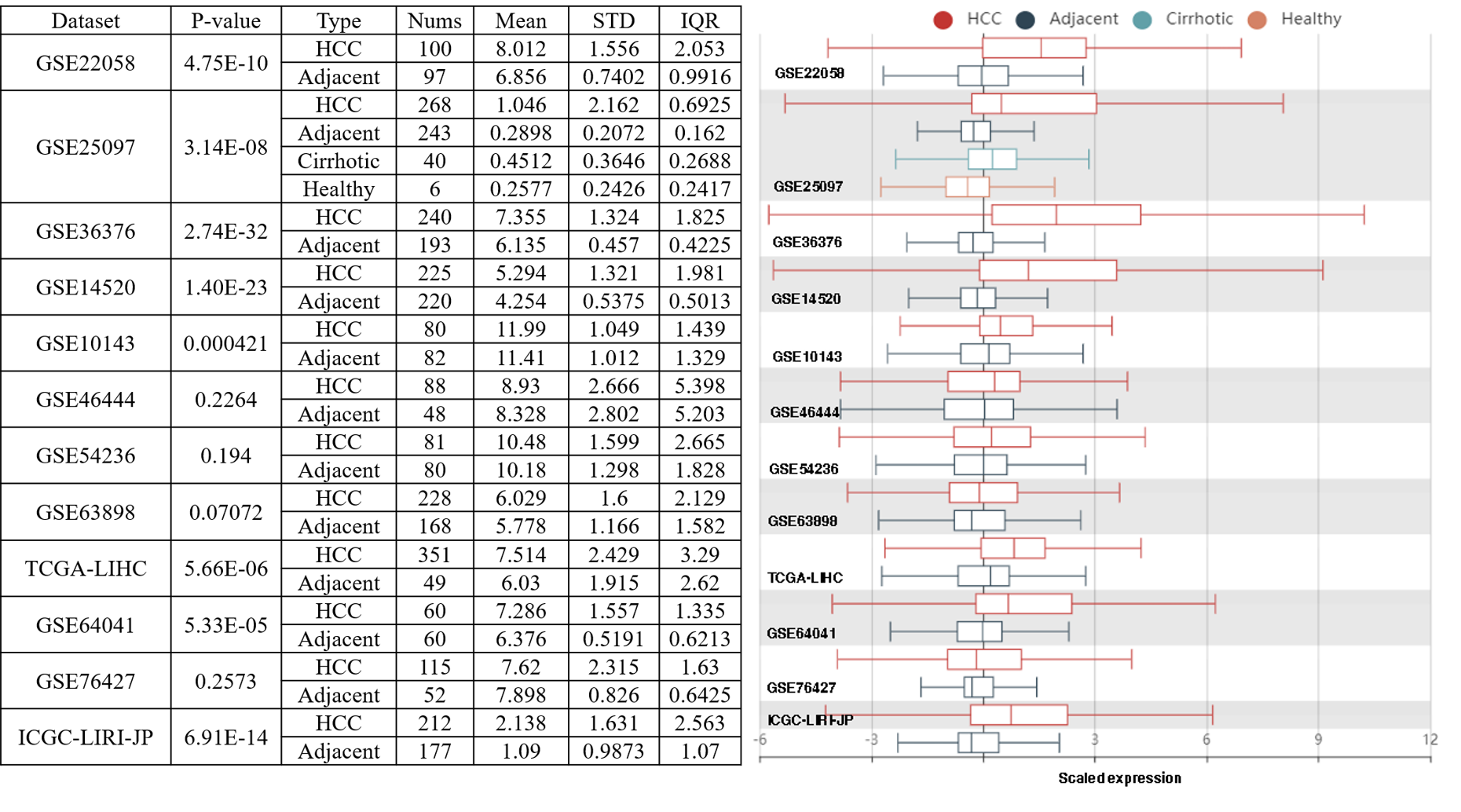

Supplement: Supplementary file 28 — Fig S28 [file JCMM-25-448-s028.tif]

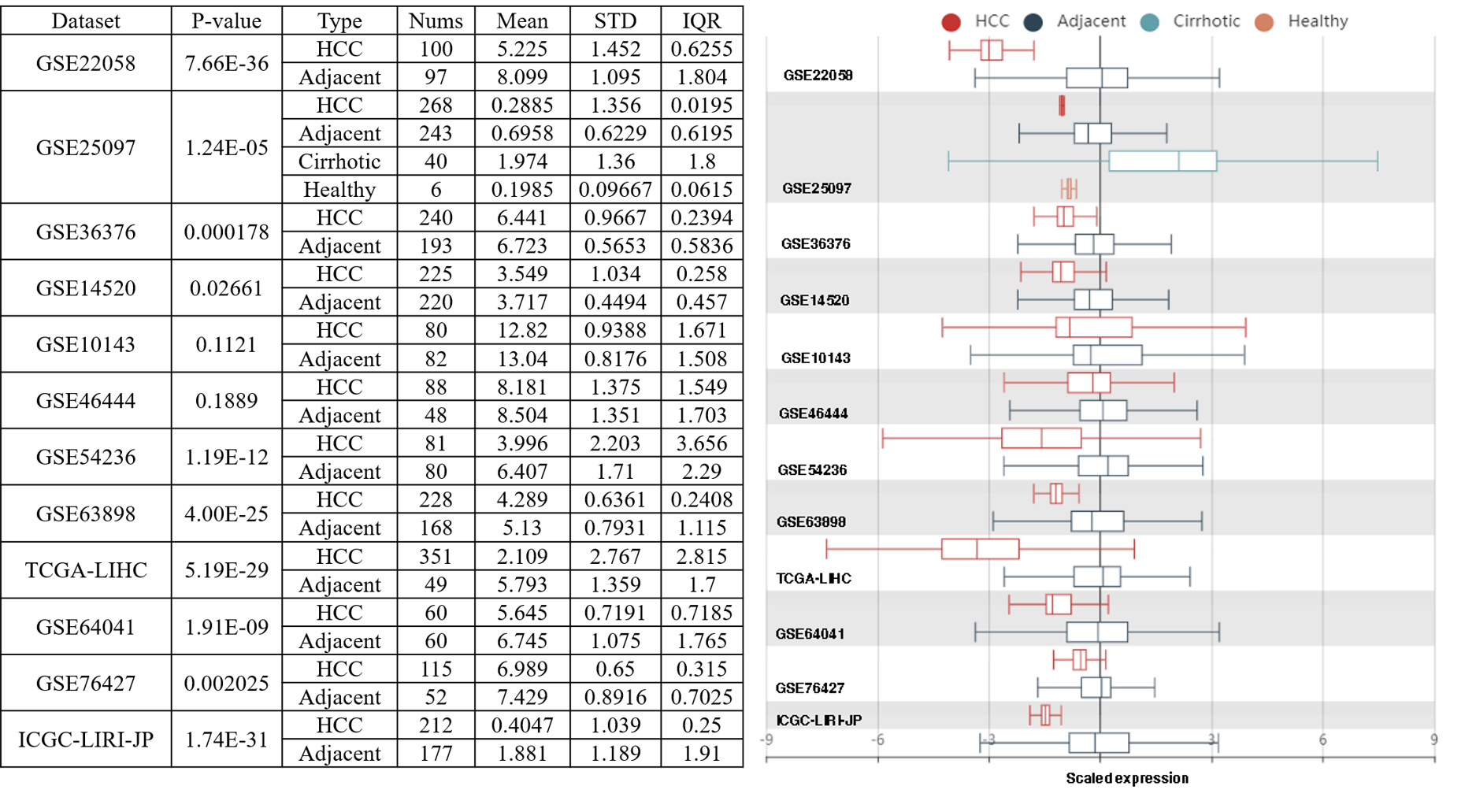

Supplement: Supplementary file 29 — Fig S29 [file JCMM-25-448-s029.tif]

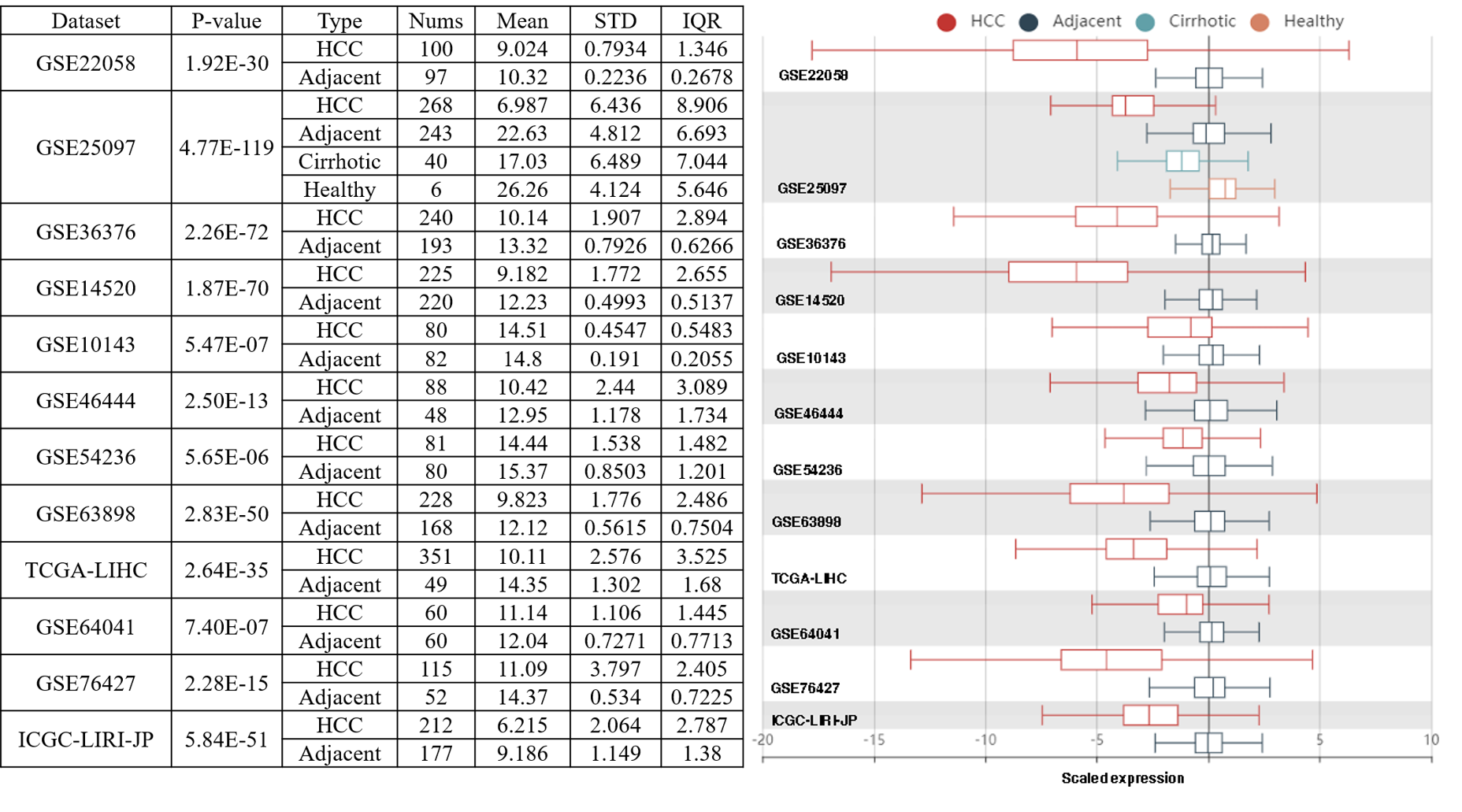

Supplement: Supplementary file 30 — Fig S30 [file JCMM-25-448-s030.tif]

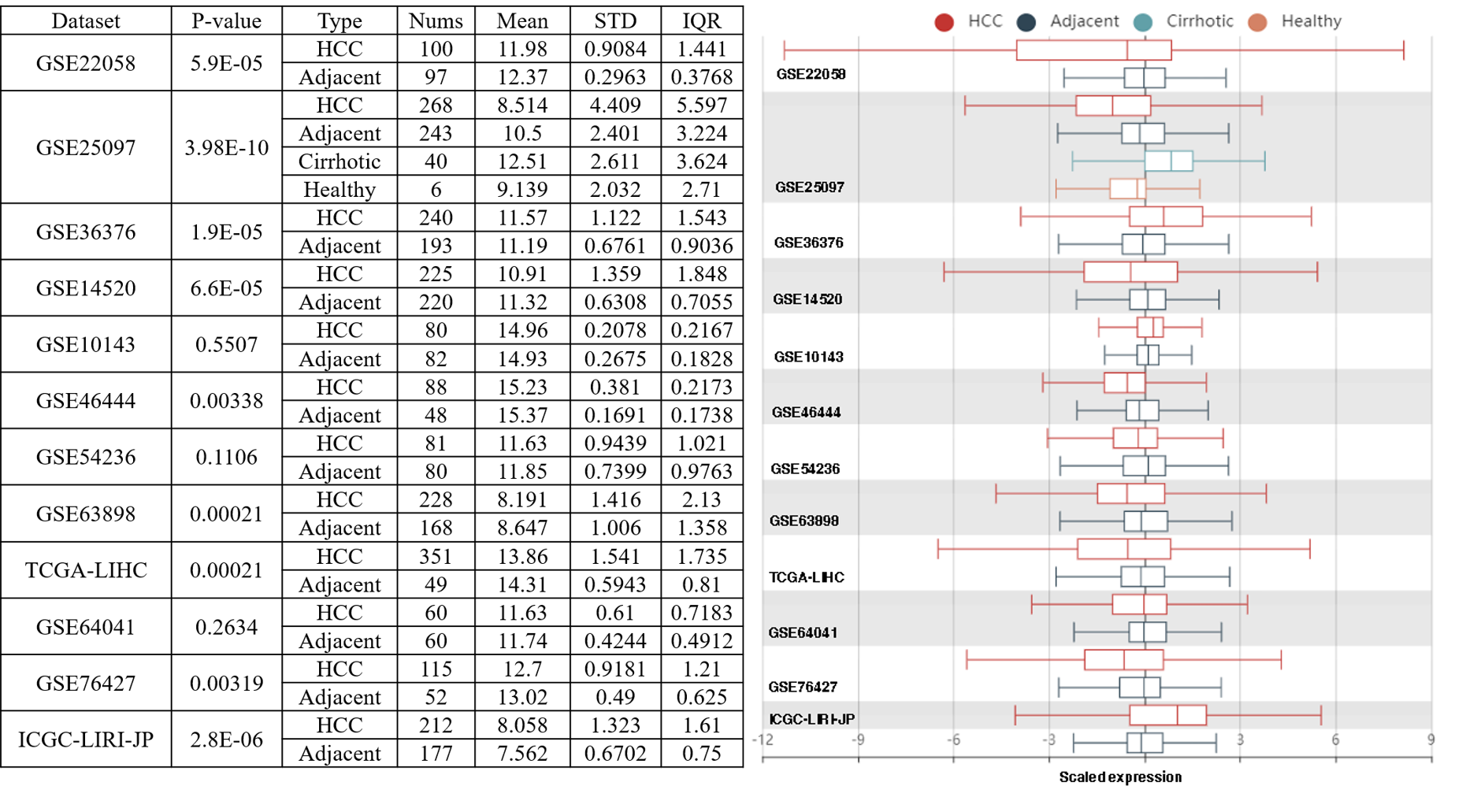

Supplement: Supplementary file 31 — Fig S31 [file JCMM-25-448-s031.tif]

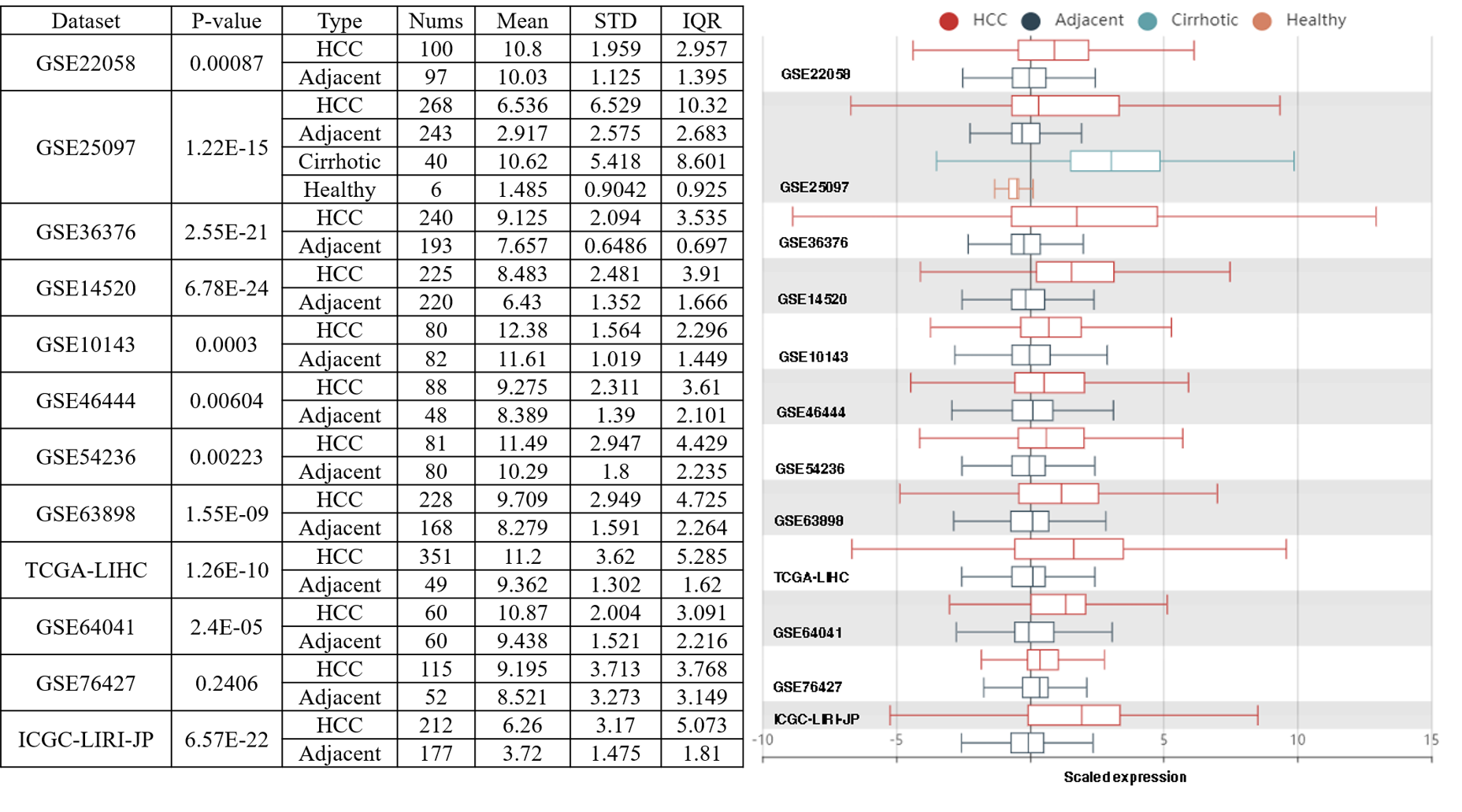

Supplement: Supplementary file 32 — Fig S32 [file JCMM-25-448-s032.tif]

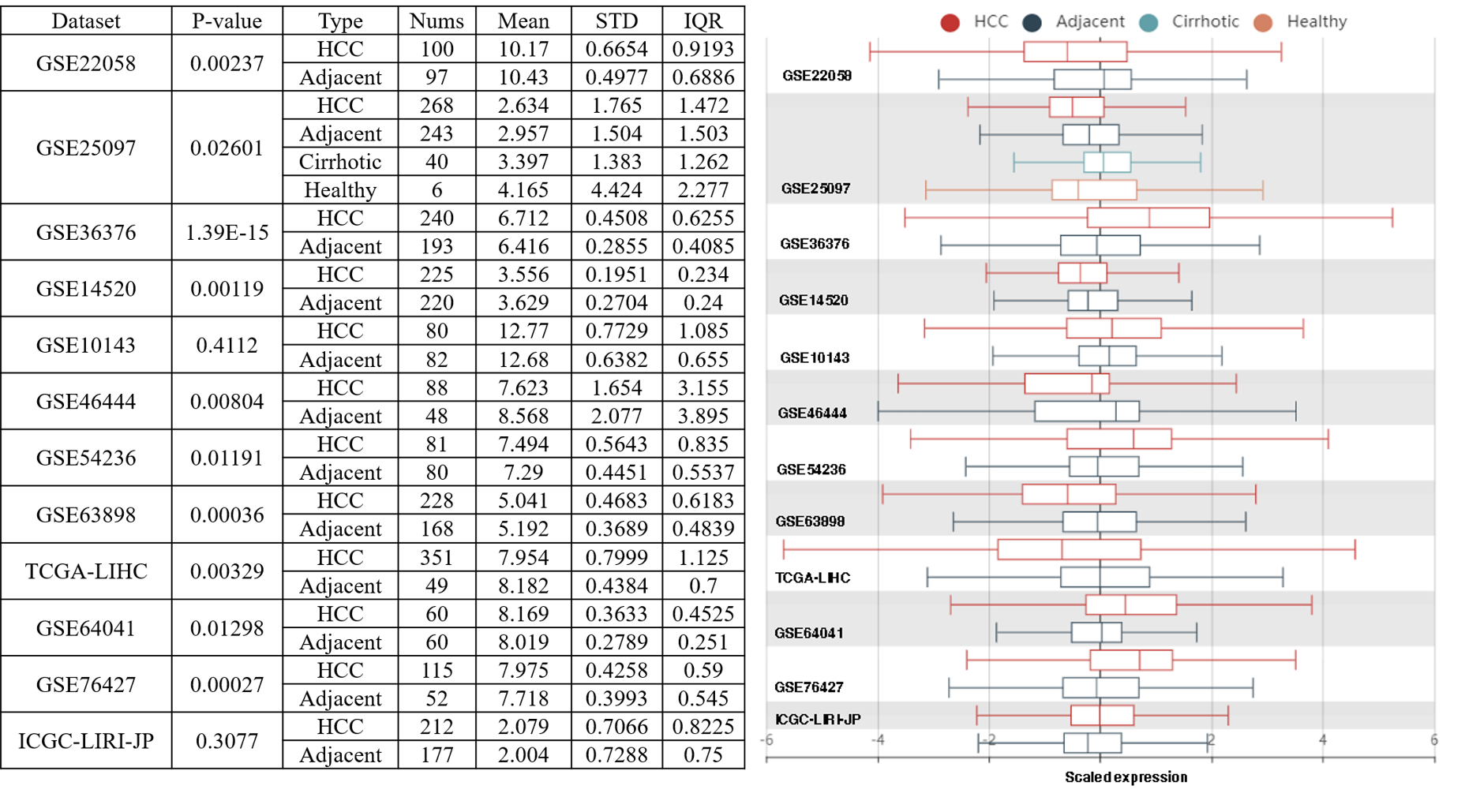

Supplement: Supplementary file 33 — Fig S33 [file JCMM-25-448-s033.tif]

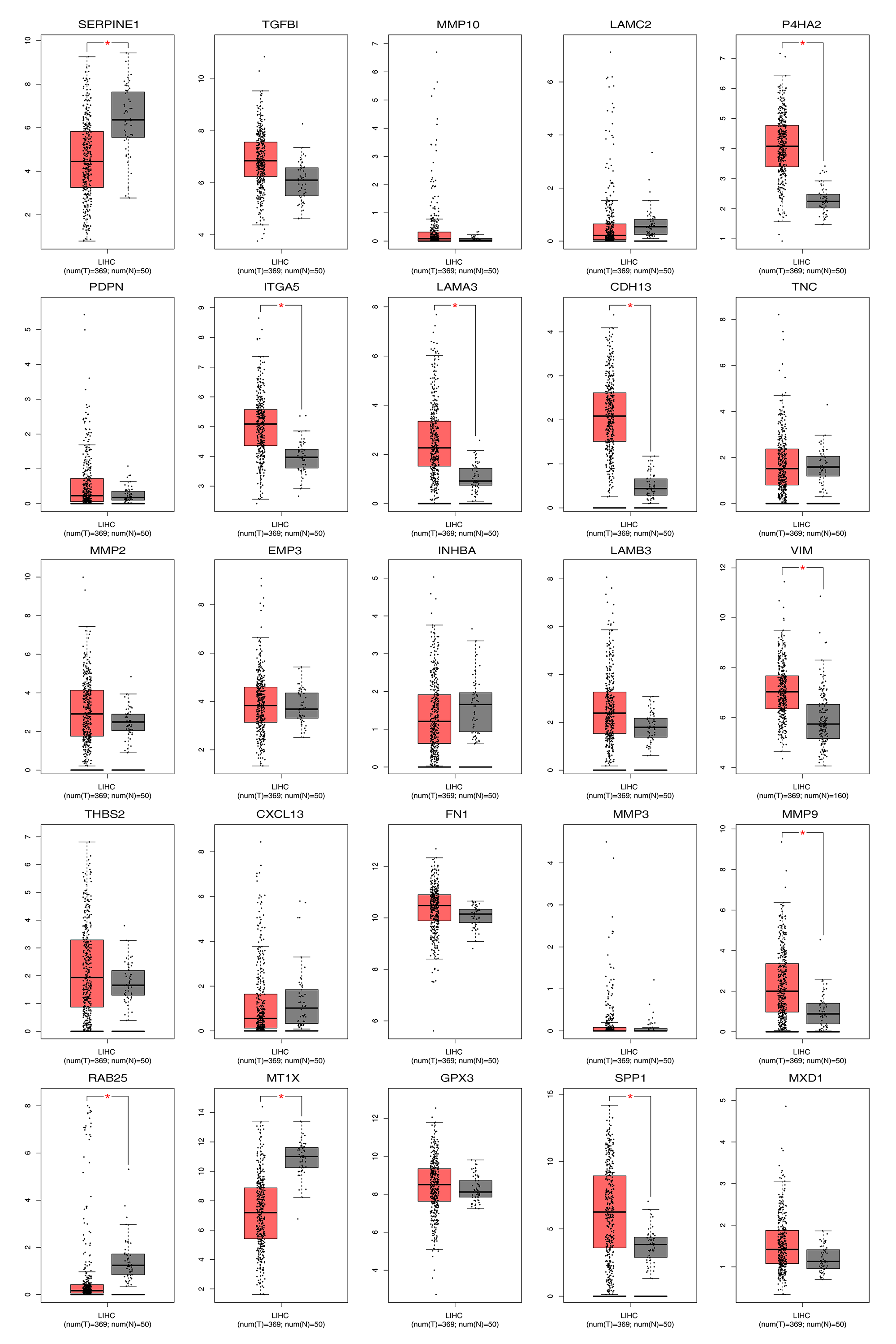

Supplement: Supplementary file 34 — Fig S34 [file JCMM-25-448-s034.tif]
