# Supplementary material for: Comprehensive analysis of partial epithelial mesenchymal transition‐related genes in hepatocellular carcinoma
Source: J Cell Mol Med. 2020 Nov 20;25(1):448–62. doi: 10.1111/jcmm.16099 (PMC7810929; doi:10.1111/jcmm.16099)
Supplement: Supplementary file 35 — Table S1 [file JCMM-25-448-s035.docx]

| Supplementary Table S1. The correlation between mRNA, miRNA and lncRNA (starBase v3.0 database) | | | |
| --- | --- | --- | --- |
| miRNA | mRNA/lncRNA | R | P Value |
| hsa-miR-148a-3p※ | ITGA5 | -0.395 | 3.07E-15 |
| hsa-miR-204-5p | MMP9 | -0.301 | 3.58E-09 |
| hsa-miR-148a-3p※ | SNHG3 | -0.268 | 1.61E-07 |
| hsa-miR-148a-3p | LINC01006 | -0.065 | 2.15E-01 |
| hsa-miR-148a-3p※ | NUTM2B-AS1 | -0.266 | 1.98E-07 |
| hsa-miR-148a-3p | TMEM132D-AS1 | -0.021 | 6.88E-01 |
| hsa-miR-148a-3p | LINC01257 | -0.012 | 8.19E-01 |
| hsa-miR-148a-3p※ | LINC00346 | -0.155 | 2.80E-03 |
| hsa-miR-148a-3p※ | SNHG20 | -0.22 | 2.02E-05 |
| hsa-miR-148a-3p | LINC00667 | 0.161 | 1.95E-03 |
| hsa-miR-148a-3p※ | LINC00909 | -0.126 | 1.50E-02 |
| hsa-miR-148a-3p※ | TUG1 | -0.24 | 3.17E-06 |
| hsa-miR-204-5p | ACVR2B-AS1 | 0.131 | 1.17E-02 |
| hsa-miR-204-5p | LINC01258 | 0.436 | 1.31E-18 |
| hsa-miR-204-5p | LINC01232 | -0.022 | 6.74E-01 |
| hsa-miR-204-5p | MCM3AP-AS1 | -0.102 | 5.01E-02 |
| ITGA5※ | SNHG3 | 0.277 | 5.46E-08 |
| ITGA5 | LINC01006 | -0.012 | 8.11E-01 |
| ITGA5※ | NUTM2B-AS1 | 0.314 | 5.17E-10 |
| ITGA5 | TMEM132D-AS1 | 0.089 | 8.42E-02 |
| ITGA5 | LINC01257 | 0.224 | 1.25E-05 |
| ITGA5※ | LINC00346 | 0.318 | 2.91E-10 |
| ITGA5※ | SNHG20 | 0.207 | 5.43E-05 |
| ITGA5 | LINC00667 | 0.046 | 3.75E-01 |
| ITGA5※ | LINC00909 | 0.296 | 5.13E-09 |
| ITGA5※ | TUG1 | 0.546 | 2.07E-30 |
| MMP9 | ACVR2B-AS1 | -0.068 | 1.91E-01 |
| MMP9 | LINC01258 | 0.014 | 7.94E-01 |
| MMP9 | LINC01232 | 0.143 | 5.62E-03 |
| MMP9 | MCM3AP-AS1 | 0.218 | 2.20E-05 |
| ※Pairs meeting the competing endogenous RNA hypothesis | | | |
